# High *EMSY* expression defines a BRCA‐like subgroup of high‐grade serous ovarian carcinoma with prolonged survival and hypersensitivity to platinum

**DOI:** 10.1002/cncr.32079

**Published:** 2019-06-02

**Authors:** Robert L. Hollis, Michael Churchman, Caroline O. Michie, Tzyvia Rye, Laura Knight, Andrena McCavigan, Timothy Perren, Alistair R. W. Williams, W. Glenn McCluggage, Richard S. Kaplan, Gordon C. Jayson, Amit Oza, D. Paul Harkin, C. Simon Herrington, Richard Kennedy, Charlie Gourley

**Affiliations:** ^1^ Nicola Murray Centre for Ovarian Cancer Research, Cancer Research UK Edinburgh Centre, Medical Research Council Institute of Genetics and Molecular Medicine University of Edinburgh Edinburgh United Kingdom; ^2^ Almac Diagnostics Craigavon United Kingdom; ^3^ St. James's Institute of Oncology St. James's University Hospital Leeds United Kingdom; ^4^ Department of Pathology University of Edinburgh Edinburgh United Kingdom; ^5^ Center for Cancer Research and Cell Biology Queen's University of Belfast Belfast United Kingdom; ^6^ Department of Pathology Belfast Health and Social Care Trust Belfast United Kingdom; ^7^ Medical Research Council Clinical Trials Unit at University College London London United Kingdom; ^8^ Division of Molecular and Clinical Cancer Sciences University of Manchester Manchester United Kingdom; ^9^ Cancer Clinical Research Unit, Division of Medical Oncology and Hematology, Princess Margaret Cancer Centre University of Toronto Toronto Ontario Canada; ^10^ Division of Pathology, Centre for Comparative Pathology, Cancer Research UK Edinburgh Centre, Medical Research Council Institute of Genetics and Molecular Medicine University of Edinburgh Edinburgh United Kingdom

**Keywords:** *EMSY*, homologous recombination, ovarian cancer, platinum response, survival

## Abstract

**Background:**

Approximately half of high‐grade serous ovarian carcinomas (HGSOCs) demonstrate homologous recombination repair (HR) pathway defects, resulting in a distinct clinical phenotype comprising hypersensitivity to platinum, superior clinical outcome, and greater sensitivity to poly(adenosine diphosphate‐ribose) polymerase (PARP) inhibitors. *EMSY*, which is known to be amplified in breast and ovarian cancers, encodes a protein reported to bind and inactivate BRCA2. Thus, *EMSY* overexpression may mimic *BRCA2* mutation, resulting in HR deficiency. However, to our knowledge, the phenotypic consequences of *EMSY* overexpression in HGSOC patients has not been explored.

**Methods:**

Here we investigate the impact of *EMSY* expression on clinical outcome and sensitivity to platinum‐based chemotherapy using available data from transcriptomically characterized HGSOC cohorts.

**Results:**

High *EMSY* expression was associated with better clinical outcome in a cohort of 265 patients with HGSOC from Edinburgh (overall survival multivariable hazard ratio, 0.58 [95% CI, 0.38‐0.88; *P *= .011] and progression‐free survival multivariable hazard ratio, 0.62 [95% CI, 0.40‐0.96; *P *= .030]). Superior outcome also was demonstrated in the Medical Research Council ICON7 clinical trial and multiple publicly available data sets. Patients within the Edinburgh cohort who had high *EMSY* expression were found to demonstrate greater rates of complete response to multiple platinum‐containing chemotherapy regimens (radiological complete response rate of 44.4% vs 12.5% at second exposure; *P *= .035) and corresponding prolonged time to disease progression (median, 151.5 days vs 60.5 days after third platinum exposure; *P *= .004).

**Conclusions:**

Patients with HGSOCs demonstrating high *EMSY* expression appear to experience prolonged survival and greater platinum sensitivity, reminiscent of *BRCA*‐mutant cases. These data are consistent with the notion that *EMSY* overexpression may render HGSOCs HR deficient.

## Abstract 2

Patients with high‐grade serous ovarian carcinomas demonstrating high expression levels of *EMSY* appear to experience prolonged survival and hypersensitivity to multiple lines of platinum‐based chemotherapy. These data support the notion that overexpression of the gene product of *EMSY*, which is reported to bind and inactivate BRCA2, may mimic the phenotype conferred by *BRCA* mutation.

## Introduction

Ovarian cancer is the most lethal gynecological malignancy, accounting for >14,000 deaths per year in the United States alone.^1^ High‐grade serous ovarian carcinoma (HGSOC) accounts for approximately 75% of cases, and is diagnosed at an advanced stage in the vast majority of patients.^2^, ^3^ Although HGSOC is typically sensitive to platinum‐based chemotherapy at the time of diagnosis, the majority of patients will experience disease recurrence, which accrues resistance to platinum resulting in sequentially shorter treatment‐free intervals before patients eventually succumb to disease.^4^

Over the last decade, a wealth of molecular data have been produced in an effort to better characterize HGSOC and to identify molecular subtypes of disease with biology that may be exploited therapeutically.^5^, ^6^, ^7^ However, currently only sequencing to detect mutations in the homologous recombination (HR) DNA repair genes *BRCA1* and *BRCA2* (*BRCA*) is routinely used to identify molecular subgroups that are clinically actionable. *BRCA*‐mutant patients experience prolonged survival, enhanced sensitivity to platinum even with multiple exposures, and a greater sensitivity to poly(adenosine diphosphate‐ribose) polymerase (PARP) inhibition by virtue of HR deficiency.^8^, ^9^, ^10^, ^11^, ^12^, ^13^

The *EMSY* gene, also known as *C11orf30*, encodes a nuclear protein that has been identified to bind and inactivate BRCA2 and is reportedly amplified in approximately 6% to 18% of HGSOC cases and 7% to 13% of sporadic breast cancer cases.^5^, ^14^, ^15^ EMSY colocalizes to sites of DNA damage, and overexpression of a truncated form of EMSY able to bind BRCA2 has been reported to induce genomic instability and sensitivity to the DNA‐damaging agent mitomycin C.^14^, ^16^ Overexpression of *EMSY* disrupts the BRCA2/RAD51 pathway after DNA damage and may override the HR players RPA and PALB2, which bind BRCA2 in the same region as EMSY.^17^ Thus, tumors with *EMSY* amplification may mimic those demonstrating mutational inactivation of *BRCA2*. Similar to *BRCA* mutation, *EMSY* amplification has been associated with poor prognosis in patients with breast cancer and is most common in the HGS histological subtype of OC.^14^, ^15^, ^18^, ^19^, ^20^, ^21^, ^22^

However, to our knowledge, no association between *EMSY* expression and clinical outcome in patients with HGSOC has been made to date. In the current study, we sought to perform in silico analysis of available transcriptomic data to investigate whether patients with HGSOCs demonstrating high expression of *EMSY* experience differential clinical outcome or sensitivity to platinum‐based chemotherapy.

## Materials and Methods

### Cohort Descriptions

The Edinburgh cohort comprised 265 HGSOC patients who were treated within the Edinburgh Cancer Centre between 1984 and 2006 and identified as part of a previous study of HGSOC.^23^ All patients received platinum‐containing first‐line chemotherapy subsequent to primary surgical debulking. The distribution of patient age at the time of diagnosis, extent of residual disease after primary debulking surgery, and International Federation of Gynecology and Obstetrics (FIGO) stage at the time of diagnosis are detailed in Table 1. The Medical Research Council (MRC) ICON7 cohort comprised 367 patients with HGSOC from the ICON7 clinical trial, 185 of whom received combination therapy with carboplatin and paclitaxel with bevacizumab and 182 of whom received the combination of carboplatin and paclitaxel alone. These specimens were from patients consenting to the translational research component of the study, and were collected across several international sites.

**Table 1:** Demographics of HGSOC Patients With High and Low *EMSY *Expression in the Edinburgh Cohort

		Low *EMSY* Expression		High *EMSY* Expression		
Demographics		No.	%	No.	%	*P*
HGSOC	Patients	228		37		
FIGO stage of disease at time of diagnosis	I	13	5.9%	1	2.8%	NS^a^
	II	15	6.8%	5	13.9%	
	III	153	68.9%	22	61.1%	
	IV	41	18.5%	8	22.2%	
	NA	6		1		
Residual disease after debulking surgery	<2 cm^b^	81	40.5%	17	48.6%	NS^c^
	2‐5 cm	51	25.5%	8	22.9%	
	>5 cm	68	34.0%	10	28.6%	
	NA	28		2		
Median age at diagnosis, years		61 (range, 32‐86)	61 (range, 43‐81)	NS^d^		

Abbreviations: FIGO, International Federation of Gynecology and Obstetrics; HGSOC, high‐grade serous ovarian carcinoma; NA, not available; NS, not significant.

a Determined by the Fisher's exact test: early‐stage (stage I‐II) versus late‐stage (stage III‐IV) disease at the time of diagnosis.

b Due to the retrospective nature of these data and the historical classification of optimal surgical resection as <2 cm residual disease in older cases, optimal surgical success could not be resolved beyond <2 cm within the Edinburgh cohort.

c Determined by the chi‐square test: <2 cm versus ≥2 cm.

d Determined by the Student *t* test.

### Edinburgh and MRC ICON7 Cohort Gene Expression Data

Gene expression data for the Edinburgh and MRC ICON7 study cohorts were generated as part of a previous study identifying transcriptomically‐defined molecular subtypes of HGSOC.^23^ Briefly, for each cohort, RNA was extracted from macrodissected, formalin‐fixed, paraffin‐embedded tumor material (High Pure kit; Roche Life Science, Indianapolis, Indiana), cDNA amplification was performed (FFPE WTA System V2; NuGEN, Leek, the Netherlands), and fragmentation and labeling was performed (NuGEN Encore Biotin Module) followed by hybridization to the Ovarian DSA cDNA microarray platform (Almac Diagnostics, Craigavon, United Kingdom). Each cohort was preprocessed using the Robust Multi‐Array Average (RMA)^24^ method prior to a comprehensive quality control analysis, including assessments of sample quality via the Affymetrix percentage present metric (Affymetrix, Santa Clara, California)^25^ and cohort metrics using principal components analysis and Kolmogorov‐Smirnov distributions analysis. Probe sets that were informative for *EMSY* gene expression were extracted and per‐sample *EMSY* expression was calculated as the mean expression between probe sets (see Supporting Table S1 and Table S2) (see Supporting Fig. S1).

### Identification of the Threshold for High EMSY Expression Within the Edinburgh Cohort

The optimal threshold for dichotomization of the Edinburgh cohort into high and low *EMSY* expression was identified using cutpoint analysis of univariable survival (see Supporting Fig. S2). This approach identified 14% as the optimal threshold, which was subsequently validated by application to independent transcriptomic data sets.

### Publicly Available Gene Expression Data Sets

Gene expression data from the studies by Pils et al,^26^ Tothill et al,^7^ Mateescu et al,^27^ and The Cancer Genome Atlas (TCGA)^5^ were accessed using the curatedOvarianData R package.^28^ *EMSY* gene expression data were extracted for samples of serous histology within their respective studies. Samples documented as serous grade 1 were excluded as possible low‐grade SOC.

### Survival Data

Clinical annotation for the Edinburgh cohort was retrieved from the Edinburgh Ovarian Cancer Database, in which data are entered prospectively in an unselected manner by a single individual as part of routine care. Survival data for the Pils et al,^26^ Tothill et al,^7^ Mateescu et al,^27^ and TCGA^5^ data sets were accessed using the curatedOvarianData R package.^28^

### Platinum Response Data

Detailed response data to each cytotoxic therapy regimen for patients in the Edinburgh cohort were collected retrospectively from the Edinburgh Ovarian Cancer Database in conjunction with archived patient notes. Radiological responses were reported as per World Health Organization or Response Evaluation Criteria in Solid Tumors (RECIST) criteria, with the exception of the need for confirmatory scans. CA 125 tumor marker responses were reported according to Gynecological Cancer InterGroup (GCIG) guidelines.^29^

### Statistical Analyses

Statistical analyses were performed using R (version 3.5.1; R Foundation, Vienna, Austria). Survival analyses were conducted using Cox proportional hazards models for progression‐free survival (PFS) and overall survival (OS). Survival differences were visualized using the Kaplan‐Meier method. Multivariable survival analyses accounted for the success of primary surgical debulking, FIGO stage at the time of diagnosis, and patient age at the time of diagnosis, with the exception of the Mateescu et al data set, in which data regarding surgical debulking and patient age were not available. Within the MRC ICON7 data set, chemotherapy regimen (bevacizumab treatment vs placebo) was also accounted for in multivariable analyses where patients from both treatment arms were analyzed together. Survival differences are presented as univariable or multivariable hazard ratios (uniHR or multiHR) alongside their corresponding 95% CIs and *P* values. Comparisons of categorical variables were performed using the chi‐square or Fisher's exact tests as appropriate. Differences in time to disease progression from receipt of platinum were evaluated using the Mann‐Whitney *U* test. Adjustments for multiple testing were made using the Bonferroni correction when specified.

## Results

### High EMSY Expression Was Associated With Superior Survival in the Edinburgh HGSOC Cohort

**Figure 1:**
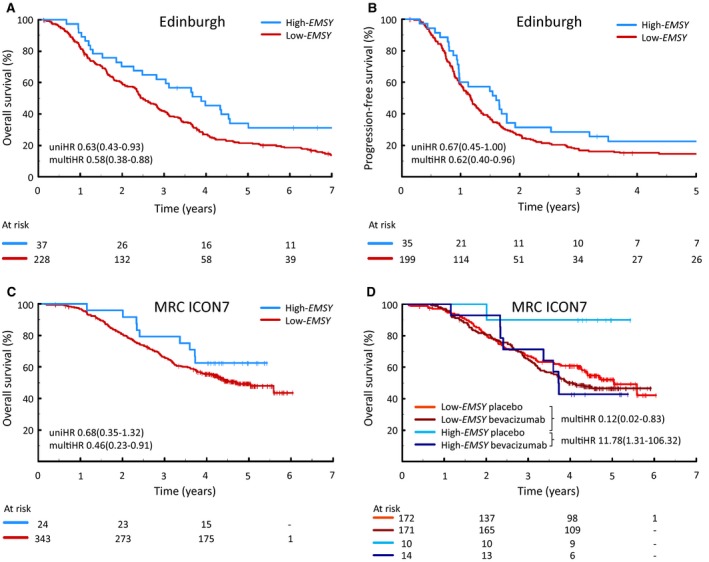
Clinical outcome in patients with high‐grade serous ovarian carcinomas (HGSOCs) demonstrating high *EMSY* expression. (A) Overall survival (OS) in the Edinburgh cohort. (B) Progression‐free survival (PFS) in the Edinburgh cohort. (C) OS in the Medical Research Council (MRC) ICON7 clinical trial cohort. (D) OS in the MRC ICON7 clinical trial cohort stratified by trial arm. multiHR indicates multivariable hazard ratio; uniHR, univariable hazard ratio.

Gene expression data for 265 HGSOCs in the Edinburgh cohort were probed for *EMSY* expression. 14% of HGSOC patients with the highest levels of *EMSY* expression (high‐*EMSY*) demonstrated prolonged OS compared with the remainder of the cohort (low‐*EMSY*) (uniHR, 0.63 [95% CI, 0.43‐0.93; *P *= .020]) (Fig. 1A) (Table 2).^5^, ^7^, ^26^, ^27^ A multivariable model accounting for FIGO stage at diagnosis, residual disease after primary debulking surgery, and patient age demonstrated an OS benefit for patients within the high‐*EMSY* group (multiHR, 0.58 [95% CI, 0.38‐0.88; *P *= .011]) (see Supporting Table S3.1). A multivariable model also demonstrated that the high‐*EMSY* group had prolonged PFS versus the low‐*EMSY* group (multiHR, 0.62 [95% CI, 0.40‐0.96; *P *= .030]) (Fig. 1B) (see Supporting Table S3.2).

**Table 2:** Univariable and Multivariable Analyses of Clinical Outcome in Patients With HGSOCs Demonstrating High *EMSY* Expression across Multiple Data Sets

				Univariable			Multivariable		
Data Set	Event Type	High EMSY Expression No. of Cases	Low EMSY Expression No. of Cases	HR	95% CI	*P*	HR	95% CI	*P*
Edinburgh	OS	37	228	0.63	0.43‐0.93	.020	0.58	0.38‐0.88	.011
	PFS			0.67	0.45‐1.00	.052	0.62	0.40‐0.96	.030
MRC ICON7 cohort	OS	24	343	0.68	0.35‐1.32	.254	0.46	0.23‐0.91	.025
	PFS			1.27	0.82‐1.97	.280	0.89	0.57‐1.38	.599
Pils et al^26^ cohort	OS	24	146	0.31	0.10‐1.02	.053	0.27	0.08‐0.87	.028
	PFS			0.70	0.41‐1.22	.210	0.52	0.29‐0.92	.026
Mateescu et al^27^cohort	OS	11	64	0.40	0.17‐0.94	.035	0.43	0.18‐0.99	.048
	PFS			0.51	0.24‐1.09	.084	0.59	0.27‐1.25	.168
Tothill et al^7^ cohort	OS	35	210	0.50	0.27‐0.93	.029	0.60	0.32‐1.13	.112
	PFS			0.70	0.44‐1.09	.114	0.63	0.39‐1.04	.072
TCGA^5^ cohort	OS	77	472	0.95	0.68‐1.34	.789	1.18	0.83‐1.66	.358
TCGA stage III/IV	PFS	71	435	0.62	0.41‐0.94	.046^a^	0.68	0.45‐1.04	.076

Abbreviations: HGSOC, high‐grade serous ovarian carcinoma; HR, hazard ratio; MRC, Medical Research Council; OS, overall survival; PFS, progression‐free survival; TCGA, The Cancer Genome Atlas.

a Bonferroni‐adjusted *P* value.

### Impact of High EMSY Expression Within the MRC ICON7 Cohort

To validate the association between superior clinical outcome and high *EMSY* expression, the gene expression cutoff value from the Edinburgh data set was applied directly to the MRC ICON7 cohort characterized on the same gene expression platform.^23^ Patients in the high‐*EMSY* group within this cohort demonstrated prolonged OS when accounting for FIGO stage at diagnosis, residual disease after primary debulking surgery, trial arm (bevacizumab‐treated patients vs control arm), and age at diagnosis (multiHR, 0.46 [95% CI, 0.23‐0.91; *P *= .025]) (Fig. 1C) (see Supporting Table S3.3), but did not demonstrate superior PFS (multiHR, 0.89 [95% CI, 0.57‐1.38; *P *= .599]) (see Supporting Fig. S3).

Despite severely limited power, analysis of the high‐*EMSY* population demonstrated that patients in the high‐*EMSY* group within the bevacizumab treatment arm (14 patients) had inferior survival compared with those patients who received chemotherapy alone (10 patients) (multiHR, 11.78 [95% CI, 1.31‐106.32; *P *= .027]) (Fig. 1D) (see Supporting Table S3.4). Within the control arm specifically, patients in the high‐*EMSY* group demonstrated markedly superior OS compared with patients in the low‐*EMSY* group (multiHR, 0.12 [95% CI, 0.02‐0.083; *P *= .032) (see Supporting Table S3.5).

### Validation of Superior Outcomes in Patients With HGSOCs With High EMSY Expression

To further validate the association between high *EMSY* expression and superior clinical outcome, publicly available gene expression data sets were accessed using the curatedOvarianData package.^28^ These data sets were characterized on a variety of platforms, thereby preventing direct application of the cutoff value from the Edinburgh cohort. Therefore, the use of thresholds at the 14th percentile of *EMSY* expression within each data set was proposed. The validity of this approach was assessed using the MRC ICON7 control cohort arm and, consistent with results using the absolute threshold derived from the Edinburgh cohort, analysis of the 14% of HGSOCs with the highest *EMSY* expression demonstrated prolonged OS (multiHR, 0.23 [95% CI, 0.07‐0.72; *P *= .012]) (see Supporting Fig. S4).

**Figure 2:**
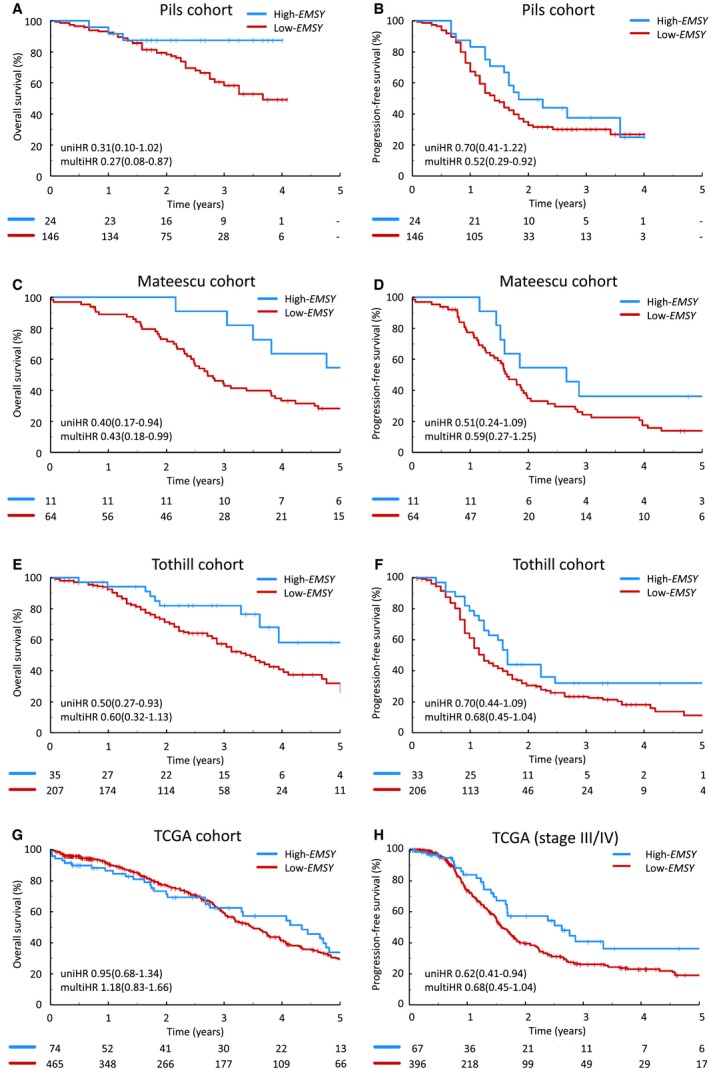
Clinical outcome of patients with high‐grade serous ovarian carcinomas (HGSOCs) demonstrating high *EMSY* expression across multiple data sets. (A) Overall survival (OS) within the Pils et al cohort.^26^ (B) Progression‐free survival (PFS) within the Pils et al cohort.^26^ (C) OS within the Mateescu et al cohort.^27^ (D) PFS within the Mateescu et al cohort.^27^ (E) OS within the Tothill et al cohort.^7^ (F) PFS within the Tothill et al cohort.^7^ (G) OS within The Cancer Genome Atlas (TCGA) cohort^5^ and (H) PFS of patients with advanced stage disease within the TCGA cohort.^5^ multiHR indicates multivariable hazard ratio; uniHR, univariable hazard ratio.

*EMSY* expression data were extracted for data sets from studies by Tothill et al,^7^ Pils et al,^26^ Mateescu et al,^27^ and TCGA.^5^ Superior OS for patients in the high‐*EMSY* group was demonstrated in the cohorts from the Pils et al^26^ (multiHR, 0.27 [95% CI, 0.08‐0.87; *P *= .028]) (Fig. 2A) (see Supporting Table S3.6) and Mateescu et al^27^ (multiHR, 0.43 [95% CI, 0.18‐0.99; *P *= .048]) (Fig. 2C) (see Supporting Table S3.7) studies. The cohort from the study by Pils et al demonstrated superior PFS within the high‐*EMSY* population (multiHR, 0.52 [95% CI, 0.29‐0.92; *P *= .026]) (Fig. 2B) (see Supporting Table S3.8).^26^ The corresponding PFS difference in the Mateescu et al^27^ cohort was not found to be statistically significant (uniHR, 0.51 [95% CI, 0.24‐1.09; *P *= .084]; and multiHR, 0.59 [95% CI, 0.27‐1.25; *P *= .168]) (Fig. 2D) (see Supporting Table S3.9). Notably, data regarding residual disease after primary debulking surgery were not available for the Mateescu et al^27^ study cohort, thereby precluding the ability to account for this variable.

Prolonged OS was demonstrated in high‐EMSY patients from the study by Tothill et al^7^ at the univariable level (uniHR, 0.50 [95% CI, 0.27‐0.93; *P *= .029]) (Fig. 2E), but did not reach statistical significance in a multivariable model accounting for patient age, FIGO stage at diagnosis, and residual disease after debulking surgery (multiHR, 0.60 [95% CI, 0.32‐1.13; *P *= .112]) (see Supporting Table S3.10). The apparent prolonged PFS noted on multivariable analysis in patients in the high‐*EMSY* group in the cohort of patients from the study by Tothill et al^7^ did not cross the threshold for statistical significance (multiHR, 0.63 [95% CI, 0.39‐1.04; *P *= .072]) (Fig. 2F) (see Supporting Table S3.11).

The TCGA cohort^5^ did not demonstrate significantly prolonged PFS within the high‐*EMSY* group (uniHR, 0.74 [95% CI, 0.50‐1.09; *P *= .122]) (see Supporting Fig. S5). PFS analysis restricted to patients diagnosed at an advanced stage of disease demonstrated prolonged PFS in this subset (uniHR, 0.62 [95% CI, 0.41‐0.94; Bonferroni‐adjusted *P *= .046]) (Fig. 2H) (see Supporting Table S3.12). High *EMSY* expression was not found to be associated with superior OS within the TCGA cohort^5^ (uniHR, 0.95 [95% CI, 0.68‐1.35]) (Fig. 2G).

### Impact of Sampling Site on the Identification of the High‐EMSY Subgroup With a Superior Clinical Outcome

Data regarding the sampling site of arrayed specimens were available for the cohort from the study by Tothill et al.^7^ Patients in the high‐*EMSY* group with sampling of the primary adnexal mass (ovary or fallopian tube) were found to demonstrate significantly superior OS (multiHR for OS, 0.28 [95% CI, 0.09‐0.90; *P *= .032]; and multiHR for PFS, 0.55 [95% CI, 0.28‐1.10; *P *= .091]) (see Supporting Figs S6A and S6C), whereas those from other sampling sites demonstrated no apparent survival benefit (multiHR for OS, 0.85 [95% CI, 0.37‐1.97; *P *= .710]; and multiHR for PFS, 0.91 [95% CI, 0.43‐1.89; *P *= .794]) (see Supporting Figs. S6B and S6D). The vast majority of samples from the Edinburgh and MRC ICON7 data sets were derived from primary adnexal masses and the sampling site data were not available for the data sets from the studies by Pils et al,^26^ Mateescu et al,^27^ and TCGA,^5^ thereby precluding investigation of the potential impact of extra‐adnexal sampling in these data sets.

### High EMSY Expression Was Associated With Greater Platinum Sensitivity in the Edinburgh Cohort

**Figure 3:**
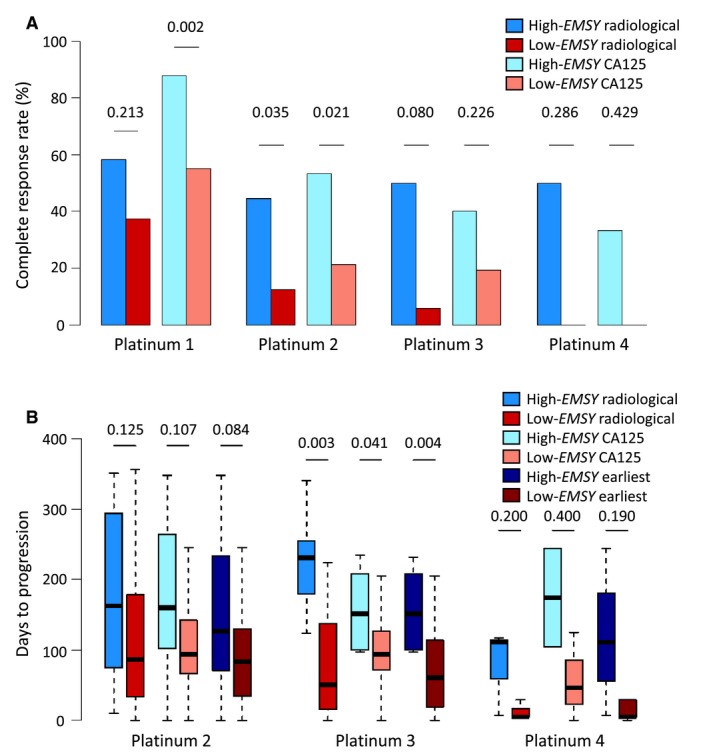
Platinum sensitivity of patients with high‐grade serous ovarian carcinomas (HGSOCs) with high *EMSY* expression within the Edinburgh cohort. (A) Rate of complete radiological and CA 125 tumor marker response to platinum‐containing chemotherapy. (B) Time to radiological, CA 125, and earliest disease progression from receipt of platinum‐containing chemotherapy.

Detailed response data regarding cytotoxic therapy regimens were collected for the Edinburgh cohort. Patients in the high‐*EMSY* group demonstrated a superior radiological complete response rate at the time of second platinum exposure (44.4% [4 of 9 patients] vs 12.5% [8 of 64 patients]; Fisher's exact *P *= .035) (Fig. 3A). Similarly, patients in the high‐*EMSY* group were found to have superior rates of complete CA 125 tumor marker response at the time of first (88.0% [22 of 25 patients] vs 55.0% [82 of 149 patients]; *P *= .002) and second (53.3% [8 of 15 patients] vs 21.3% [17 of 80 patients]; *P *= .021) platinum exposure. At the time of fourth platinum exposure, patients in the high‐*EMSY* group demonstrated a significantly greater objective CA 125 response rate (100% [3 of 3 patients] vs 0% [0 of 4 patients]; Fisher's exact *P *= .029) (see Supporting Fig. S7). Response data stratified by type of platinum‐containing regimen are detailed in Supporting Table S4.

The median time to first (radiological or CA 125 tumor marker) disease progression after second platinum exposure was 127 days in the high‐*EMSY* group compared with 83.5 days in the low‐*EMSY* group, but this did not reach statistical significance (*P *= .084) (Fig. 3B). Patients in the high‐*EMSY* group demonstrated a significantly longer time to first disease progression after third platinum exposure (median, 151.5 days vs 60.5 days; *P *= .004), which was significant when considering only progression by radiology (median, 231 days vs 50 days; *P *= .003) and CA 125 tumor marker (median, 151.5 days vs 94 days; *P *= .041) specifically.

### High EMSY Expression Was Associated With Superior Outcome in Patients With High‐Risk HGSOC

Patients with advanced stage (FIGO stage III/IV) HGSOC with gross macroscopic residual disease after debulking surgery have particularly poor prognosis (“high‐risk” patients).^30^ Within the Edinburgh cohort, a greater percentage of patients in the high‐*EMSY* group remained alive without disease recurrence within the context of high‐risk disease at 2 years (25.0% [4 of 16 patients] vs 9.2% [10 of 109 patients]; *P *= .081), 3 years (18.8% [3 of 16 patients] vs 3.6% [4 of 110 patients]; *P *= .043), 5 years (17.6% [3 of 17 patients] vs 2.7% [3 of 111 patients]; *P *= .031), and 10 years (12.5% [2 of 16 patients] vs 0.9% [1 of 112 patients]; *P *= .041) from diagnosis (see Supporting Fig. S8A), suggesting that high‐risk patients with high *EMSY* expression are more likely to achieve favorable long‐term clinical outcome.

A similar effect was observed within the cohort from the study by Pils et al^26^ at 12 months (100% [9 of 9 patients] vs 61.5% [24 of 39 patients]; *P *= .041), 18 months (88.9% [8 of 9 patients] vs 17.6% [6 of 34 patients]; *P *< .001), and 2 years (50.0% [4 of 8 patients] vs 3.1% [1 of 32 patients]; *P *= .004) (see Supporting Fig. S8B). Although similar trends were observed in the high‐risk patients with adnexal specimens from the study by Tothill et al^7^ at 12 months from diagnosis, these did not approach statistical significance (100% [5 of 5 patients] vs 62.5% [20 of32 patients]; *P *= .152) (see Supporting Fig. S9). However, patients with late‐stage disease in the Tothill et al^7^ study cohort (irrespective of residual disease after debulking surgery) demonstrated the same effect at 12 months (95% [19 of 20 patients] vs 67.8% [78 of 115 patients]; *P *= .014) and 18 months (70% [14 of 20 patients] vs 42.3% [47 of 111 patients]; *P *= .029) from diagnosis (see Supporting Fig. S8C).

## Discussion

Approximately one‐half of HGSOCs are described as having identifiable defects in the HR pathway, with the archetypal defects being germline or somatic *BRCA* inactivation.^3^ Surprisingly, the *EMSY* gene, the product of which has been shown to bind and inactivate BRCA2, has received relatively little attention in HGSOC, despite being associated with a poor prognosis in individuals with breast cancer.^14^, ^15^, ^18^, ^19^, ^20^, ^21^, ^22^

Amplification of 11q13 has been identified as a common event in patients with breast cancer and OC, and previous investigations have pointed toward *EMSY* as the critical gene in this amplicon.^14^, ^15^, ^22^ To our knowledge, only a single study to date has reported on 11q13 amplification and prognosis in patients with OC, and reported no survival difference in patients with SOC upon multivariable analysis,^21^ with other studies not investigating the impact on patient outcome.^5^, ^15^ Notably, this study did not distinguish HGSOC from low‐grade SOC, which now is recognized as a distinct clinical and molecular disease entity.^31^, ^32^, ^33^ Moreover, given the mixed reports of correlation strength between *EMSY* copy number and expression,^14^, ^15^, ^34^ investigation of the association between *EMSY* expression and survival may prove more fruitful than associations with copy number alone.

Through in silico analysis of local and publicly available transcriptomic data, we identify a subgroup of HGSOCs defined by high levels of *EMSY* expression. The threshold for *EMSY* overexpression was defined within the Edinburgh cohort and validated directly within the MRC ICON7 cohort characterized on the same platform. To identify the high‐*EMSY* population within independent cohorts characterized by heterogeneous methodologies, we used a percentile‐based expression threshold after validating this approach in the MRC ICON7 cohort. Although a significant difference in outcome within the TCGA cohort was evident only upon exploratory analysis limited to patients with advanced stage disease at the time of diagnosis, multivariable analysis restricted to patients with late‐stage disease in the other evaluable cohorts confirmed prolonged survival in their respective populations (data not shown).

Similar to *BRCA*‐mutant HGSOC, high‐*EMSY* HGSOCs appear to demonstrate prolonged survival across multiple independent data sets, and demonstrate a greater benefit from platinum‐based chemotherapy than their low‐*EMSY* counterparts. Patients in the high‐*EMSY* group within the Edinburgh cohort demonstrated a >3‐fold radiological complete response rate to second platinum exposure compared with patients in the low‐*EMSY* group.

Intriguingly, within the MRC ICON7 cohort, we demonstrated that the benefit conferred by high *EMSY* expression may be abrogated upon addition of the antiangiogenic agent bevacizumab to first‐line therapy, although the power of these analyses was severely limited. Clearly, overinterpretation of these data must be avoided in light of the low numbers of patients in the high‐*EMSY* group between the two treatment arms, and the presented analyses do not fulfil the REporting recommendations for tumor MARKer prognostic studies (REMARK) criteria for biomarker studies.^35^ However, these data do suggest that analysis of the differential impact of bevacizumab treatment between HR‐intact and HR‐deficient HGSOCs may now be warranted. Robust evaluation of the impact of HR status on bevacizumab efficacy has the potential to better define those patients who are most likely to derive benefit from the addition of antiangiogenic agents to first‐line care. These data will be of particular interest in light of ongoing trials combining antiangiogenics with PARP inhibitors.^36^

Advanced stage of disease at the time of diagnosis and suboptimal surgical resection both are associated with markedly inferior survival in patients with HGSOC.^30^ Patients with both of these features represent those with particularly high‐risk disease. We observed that within these high‐risk patients, those with high *EMSY* expression have a greater chance of remaining free of disease recurrence (12.5% vs <1% at 10 years from diagnosis within the Edinburgh cohort), suggesting that patients in the high‐*EMSY* group could represent a group with favorable long‐term clinical outcome even in the face of otherwise poor prognostic markers.

Within the data set from the study by Tothill et al,^7^ we observed that although high *EMSY* expression was associated with prolonged survival, this phenomenon was not apparent in patients in whom expression data were generated using samples that were not taken from the primary adnexal mass. There are several plausible explanations for this observation. First, differences in the tumor microenvironment at different anatomic sites may well have impacted *EMSY* expression. Second, the contamination of tumor samples with different nonmalignant cell types has the potential to alter bulk *EMSY* expression. Third, intratumor heterogeneity as a result of tumor evolution may have resulted in differential tumor cell *EMSY* expression between the primary tumor mass and more distant disease sites. In any respect, differential gene expression between sampling sites has clear implications for the identification of pertinent molecular events in both the clinical and research setting. In particular, the frequency of extra‐adnexal sampling may account for weakened trends between *EMSY* expression and clinical outcome in some data sets. Transcriptomic characterization of the Edinburgh^23^ and MRC ICON7 cohorts was performed on specimens that were nearly exclusively taken from the primary adnexal mass, and in both of these data sets samples were macrodissected prior to RNA extraction to minimize stromal contamination. Both these cohorts demonstrated a marked OS benefit in patients in the high‐*EMSY* group.

These data demonstrate the power of in silico analysis of preexisting data sets to identify novel, clinically meaningful subtypes of disease. Collectively, the current study findings allude to a subgroup of HGSOC patients defined by high levels of *EMSY* expression who appear to demonstrate improved clinical outcome and greater sensitivity to platinum‐based chemotherapy, consistent with the notion that *EMSY* overexpression renders HGSOCs HR deficient. Given the role of EMSY in disrupting the BRCA2/RAD51 HR pathway demonstrated by previous studies^14^, ^16^, ^17^ and the apparent *BRCA*‐like clinical phenotype described here in patients with high‐*EMSY* HGSOC, investigation of the sensitivity of *EMSY*‐overexpressing tumors to PARP inhibition has the potential to improve our understanding of which patients benefit the most from these agents. The correlation of *EMSY* expression with existing HR deficiency signatures and scores should also be explored. Moreover, the impact of differential *EMSY* expression in other disease settings, most notably breast and prostate cancer, should now be investigated.

## Funding Support

Funded by a Medical Research Council PhD Studentship and Medical Research Council‐funded Research Fellowship awarded to Robert L. Hollis, and by charitable donation from The Nicola Murray Foundation.

## Conflict of Interest Disclosures

Laura Knight, Andrena McCavigan, D. Paul Harkin, and Richard Kennedy are employees of Almac Diagnostics, a precision medicine company focused on the discovery, validation, and commercialization of novel diagnostic and companion diagnostic tests. Gordon C. Jayson has received grants from Roche and AstraZeneca for work performed outside of the current study. Charlie Gourley has received honoraria from AstraZeneca, Tesaro, Cor2Ed, Medscape, and Sierra Oncology; has acted as a paid consultant for AstraZeneca, Clovis, NuCana, Tesaro, Roche, Foundation One, and Cor2Ed; and is named on 1 patent issued (patent PCT/US2012/040805) and 4 pending patents (patent PCT/GB2013/053202, patent 1409479.1, patent 1409476.7, and patent 1409478.3) related to gene expression signatures predicting treatment response in ovarian cancer and his institution receives research funding from AstraZeneca, Aprea AB, NuCana, and Tesaro for work performed outside of the current study. The other authors made no disclosures.

## Author Contributions

**Robert L. Hollis:** Conceptualization, data curation, formal analysis, investigation, visualization, writing–original draft, and writing–review and editing. **Michael Churchman:** Conceptualization and writing–review and editing. **Caroline O. Michie:** Investigation. **Tzyvia Rye: **Data curation. **Laura Knight** and **Andrena McCavigan:** Methodology. **Timothy Perren**, **Gordon C. Jayson**, **Richard S. Kaplan**, **Amit Oza**, **D. Paul Harkin**, and **Richard Kennedy:** Resources and writing–review and editing. **Alistair Williams** and **W. Glenn McCluggage:** Investigation and writing–review and editing. **C. Simon Herrington:** Investigation, supervision, conceptualization, and writing–review and editing. **Charlie Gourley:** Conceptualization, supervision, funding acquisition, writing–original draft, and writing–review and editing.

## References

[1] Siegel RL , Miller KD , Jemal A . Cancer statistics, 2016. CA Cancer J Clin. 2016;66:7‐30. 26742998 10.3322/caac.21332

[2] Kurman RJ . Origin and molecular pathogenesis of ovarian high‐grade serous carcinoma. Ann Oncol. 2013;24(suppl 10):x16‐x21. 24265397 10.1093/annonc/mdt463

[3] Bowtell DD , Bohm S , Ahmed AA , et al. Rethinking ovarian cancer II: reducing mortality from high‐grade serous ovarian cancer. Nat Rev Cancer. 2015;15:668‐679. 26493647 10.1038/nrc4019 PMC4892184

[4] Cooke SL , Brenton JD . Evolution of platinum resistance in high‐grade serous ovarian cancer. Lancet Oncol. 2011;12:1169‐1174. 21742554 10.1016/S1470-2045(11)70123-1

[5] Cancer Genome Atlas Research Network . Integrated genomic analyses of ovarian carcinoma. Nature. 2011;474:609‐615. 21720365 10.1038/nature10166 PMC3163504

[6] Patch AM , Christie EL , Etemadmoghadam D , et al. Whole‐genome characterization of chemoresistant ovarian cancer. Nature. 2015;521:489‐494. 26017449 10.1038/nature14410

[7] Tothill RW , Tinker AV , George J , et al. Novel molecular subtypes of serous and endometrioid ovarian cancer linked to clinical outcome. Clin Cancer Res. 2008;14:5198‐5208. 18698038 10.1158/1078-0432.CCR-08-0196

[8] Tan DS , Rothermundt C , Thomas K , et al. “BRCAness” syndrome in ovarian cancer: a case‐control study describing the clinical features and outcome of patients with epithelial ovarian cancer associated with BRCA1 and BRCA2 mutations. J Clin Oncol. 2008;26:5530‐5536. 18955455 10.1200/JCO.2008.16.1703

[9] Candido‐dos‐Reis FJ , Song H , Goode EL , et al; for EMBRACE ; kConFab Investigators ; Australian Ovarian Cancer Study Group . Germline mutation in BRCA1 or BRCA2 and ten‐year survival for women diagnosed with epithelial ovarian cancer. Clin Cancer Res. 2015;21:652‐657. 25398451 10.1158/1078-0432.CCR-14-2497 PMC4338615

[10] Ledermann J , Harter P , Gourley C , et al. Olaparib maintenance therapy in platinum‐sensitive relapsed ovarian cancer. N Engl J Med. 2012;366:1382‐1392. 22452356 10.1056/NEJMoa1105535

[11] Fong PC , Boss DS , Yap TA , et al. Inhibition of poly(ADP‐ribose) polymerase in tumors from BRCA mutation carriers. N Engl J Med. 2009;361:123‐134. 19553641 10.1056/NEJMoa0900212

[12] Bolton KL , Chenevix‐Trench G , Goh C , et al;EMBRACE ; kConFab Investigators ; Cancer Genome Atlas Research Network . Association between BRCA1 and BRCA2 mutations and survival in women with invasive epithelial ovarian cancer. JAMA. 2012;307:382‐390. 22274685 10.1001/jama.2012.20 PMC3727895

[13] Farmer H , McCabe N , Lord CJ , et al. Targeting the DNA repair defect in BRCA mutant cells as a therapeutic strategy. Nature. 2005;434:917‐921. 15829967 10.1038/nature03445

[14] Hughes‐Davies L , Huntsman D , Ruas M , et al. EMSY links the BRCA2 pathway to sporadic breast and ovarian cancer. Cell. 2003;115:523‐535. 14651845 10.1016/s0092-8674(03)00930-9

[15] Brown LA , Irving J , Parker R , et al. Amplification of EMSY, a novel oncogene on 11q13, in high grade ovarian surface epithelial carcinomas. Gynecol Oncol. 2006;100:264‐270. 16236351 10.1016/j.ygyno.2005.08.026

[16] Raouf A , Brown L , Vrcelj N , et al. Genomic instability of human mammary epithelial cells overexpressing a truncated form of EMSY. J Natl Cancer Inst. 2005;97:1302‐1306. 16145051 10.1093/jnci/dji254

[17] Cousineau I , Belmaaza A . EMSY overexpression disrupts the BRCA2/RAD51 pathway in the DNA‐damage response: implications for chromosomal instability/recombination syndromes as checkpoint diseases. Mol Genet Genomics. 2011;285:325‐340. 21409565 10.1007/s00438-011-0612-5 PMC3064890

[18] Brown LA , Johnson K , Leung S , et al. Co‐amplification of CCND1 and EMSY is associated with an adverse outcome in ER‐positive tamoxifen‐treated breast cancers. Breast Cancer Res Treat. 2010;121:347‐354. 19636701 10.1007/s10549-009-0479-x

[19] Kirkegaard T , Nielsen KV , Jensen LB , et al. Genetic alterations of CCND1 and EMSY in breast cancers. Histopathology. 2008;52:698‐705. 18393977 10.1111/j.1365-2559.2008.03007.x

[20] Zhu Y , Wu J , Zhang C , et al. BRCA mutations and survival in breast cancer: an updated systematic review and meta‐analysis. Oncotarget. 2016;7:70113‐70127. 27659521 10.18632/oncotarget.12158 PMC5342539

[21] Brown LA , Kalloger SE , Miller MA , et al. Amplification of 11q13 in ovarian carcinoma. Genes Chromosomes Cancer. 2008;47:481‐489. 18314909 10.1002/gcc.20549

[22] Rodriguez C , Hughes‐Davies L , Valles H , et al. Amplification of the BRCA2 pathway gene EMSY in sporadic breast cancer is related to negative outcome. Clin Cancer Res. 2004;10:5785‐5791. 15355907 10.1158/1078-0432.CCR-03-0410

[23] Gourley C , McCavigan A , Perren T , et al. Molecular subgroup of high‐grade serous ovarian cancer (HGSOC) as a predictor of outcome following bevacizumab. J Clin Oncol. 2014;32:5502.

[24] Irizarry RA , Bolstad BM , Collin F , Cope LM , Hobbs B , Speed TP . Summaries of Affymetrix GeneChip probe level data. Nucleic Acids Res. 2003;31:e15. 12582260 10.1093/nar/gng015 PMC150247

[25] McCall MN , Murakami PN , Lukk M , Huber W , Irizarry RA . Assessing affymetrix GeneChip microarray quality. BMC Bioinformatics. 2011;12:137. 21548974 10.1186/1471-2105-12-137 PMC3097162

[26] Pils D , Hager G , Tong D , et al. Validating the impact of a molecular subtype in ovarian cancer on outcomes: a study of the OVCAD Consortium. Cancer Sci. 2012;103:1334‐1341. 22497737 10.1111/j.1349-7006.2012.02306.x PMC7659264

[27] Mateescu B , Batista L , Cardon M , et al. miR‐141 and miR‐200a act on ovarian tumorigenesis by controlling oxidative stress response. Nat Med. 2011;17:1627‐1635. 22101765 10.1038/nm.2512

[28] Ganzfried BF , Riester M , Haibe‐Kains B , et al. curatedOvarianData: clinically annotated data for the ovarian cancer transcriptome. Database (Oxford). 2013;2013:bat013. 23550061 10.1093/database/bat013 PMC3625954

[29] Rustin GJ , Vergote I , Eisenhauer E , et al; Gynecological Cancer Intergroup . Definitions for response and progression in ovarian cancer clinical trials incorporating RECIST 1.1 and CA 125 agreed by the Gynecological Cancer Intergroup (GCIG). Int J Gynecol Cancer. 2011;21:419‐423. 21270624 10.1097/IGC.0b013e3182070f17

[30] Hoppenot C , Eckert MA , Tienda SM , Lengyel E . Who are the long‐term survivors of high grade serous ovarian cancer? Gynecol Oncol. 2018;148:204‐212. 29128106 10.1016/j.ygyno.2017.10.032

[31] Gockley A , Melamed A , Bregar AJ , et al. Outcomes of women with high‐grade and low‐grade advanced‐stage serous epithelial ovarian cancer. Obstet Gynecol. 2017;129:439‐447. 28178043 10.1097/AOG.0000000000001867 PMC5328143

[32] Prat J . New insights into ovarian cancer pathology. Ann Oncol. 2012;23(suppl 10):x111‐x117. 22987944 10.1093/annonc/mds300

[33] Hollis RL , Gourley C . Genetic and molecular changes in ovarian cancer. Cancer Biol Med. 2016;13:236‐247. 27458531 10.20892/j.issn.2095-3941.2016.0024 PMC4944549

[34] Wilkerson PM , Dedes KJ , Wetterskog D , et al. Functional characterization of EMSY gene amplification in human cancers. J Pathol. 2011;225:29‐42. 21735447 10.1002/path.2944

[35] McShane LM , Altman DG , Sauerbrei W , Taube SE , Gion M , Clark GM ; Statistics Subcommittee of the NCI‐EORTC Working Group on Cancer Diagnostics . REporting recommendations for tumour MARKer prognostic studies (REMARK). Br J Cancer. 2005;93:387‐391. 16106245 10.1038/sj.bjc.6602678 PMC2361579

[36] Mirza MR , Mortensen CE , Avall‐Lundqvist E , et al. ENGOT‐OV24‐NSGO/AVANOVA: niraparib versus bevacizumab‐niraparib combination versus bevacizumab and niraparib as sequential therapy in women with platinum‐sensitive epithelial ovarian, fallopian tube, or peritoneal cancer. J Clin Oncol. 2015;33:TPS5607.

