# Supplementary figures and images for: High EMSY expression defines a BRCA‐like subgroup of high‐grade serous ovarian carcinoma with prolonged survival and hypersensitivity to platinum
Source: Cancer. 2019 Jun 2;125(16):2772–81. doi: 10.1002/cncr.32079 (PMC6771827; doi:10.1002/cncr.32079)

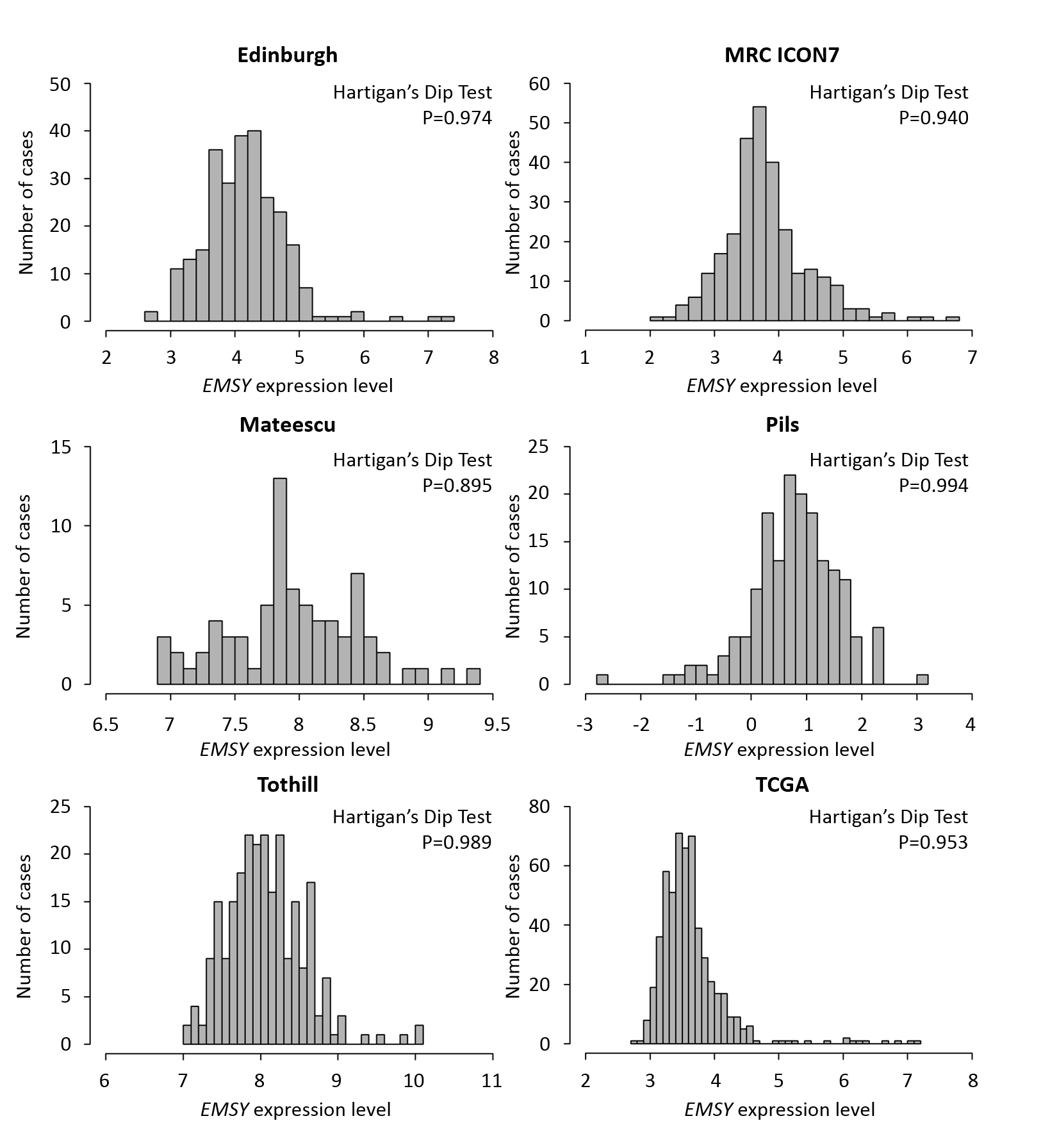

Supplement: Supplementary file 1 [file CNCR-125-2772-s001.tif]

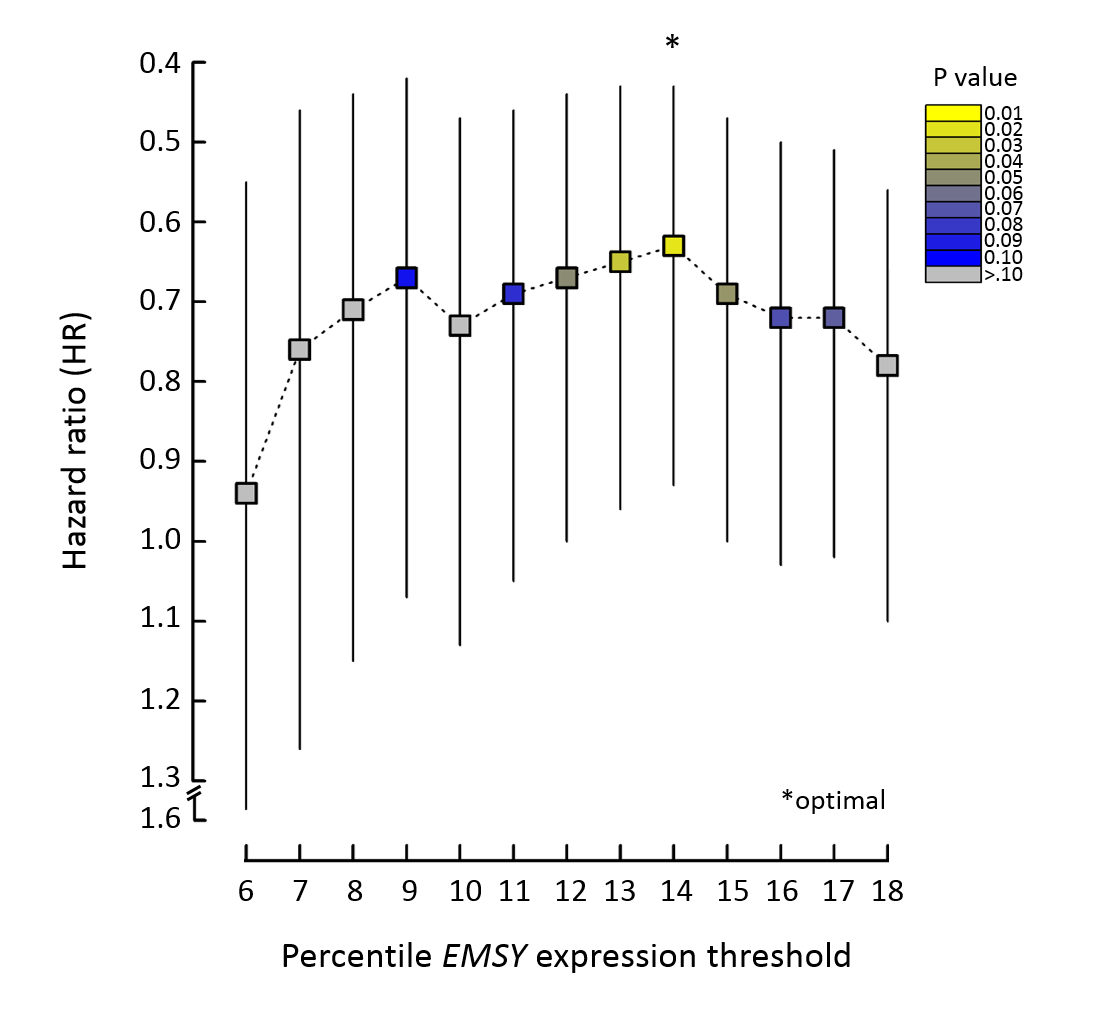

Supplement: Supplementary file 2 [file CNCR-125-2772-s002.tif]

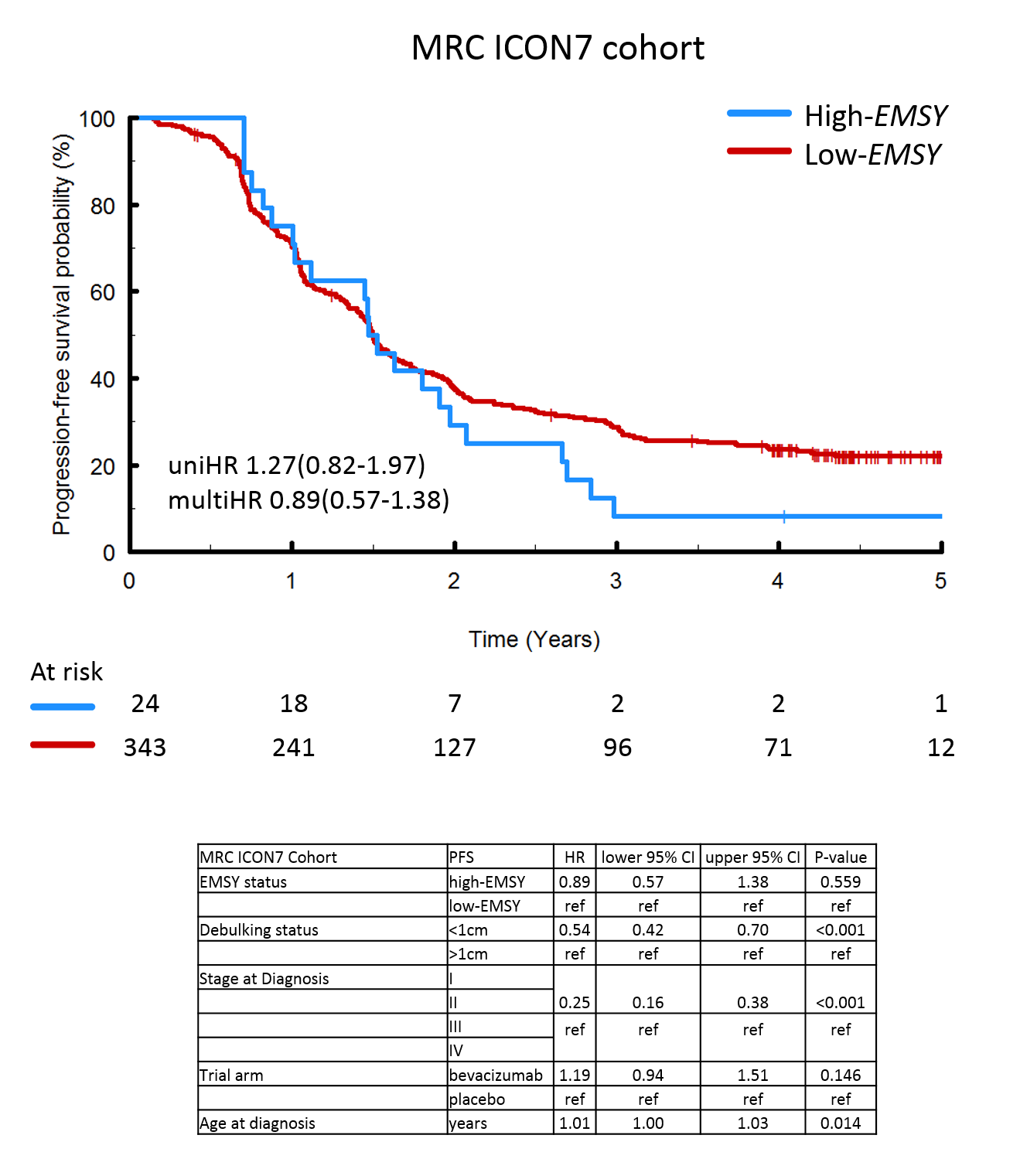

Supplement: Supplementary file 3 [file CNCR-125-2772-s003.tif]

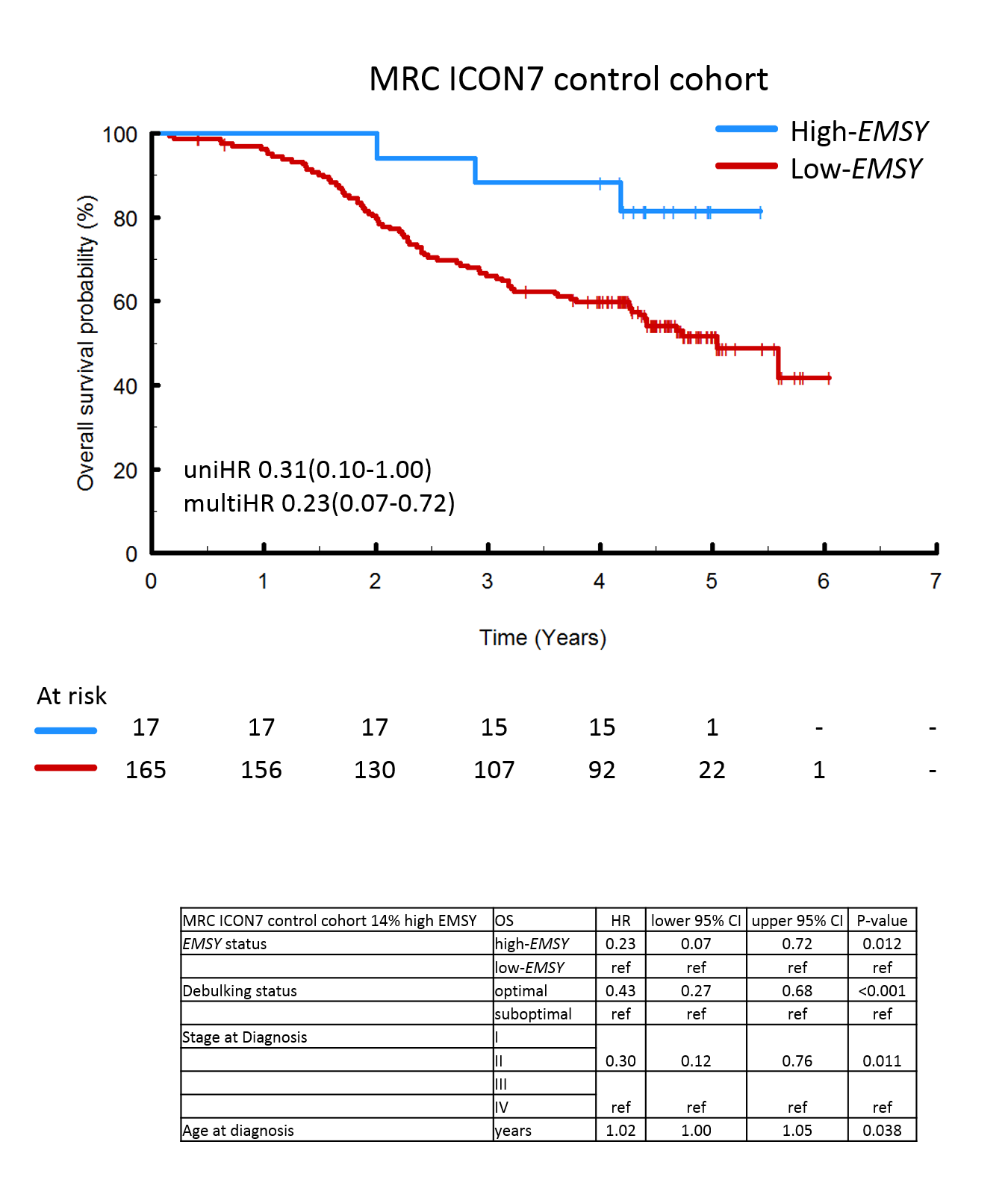

Supplement: Supplementary file 4 [file CNCR-125-2772-s004.tif]

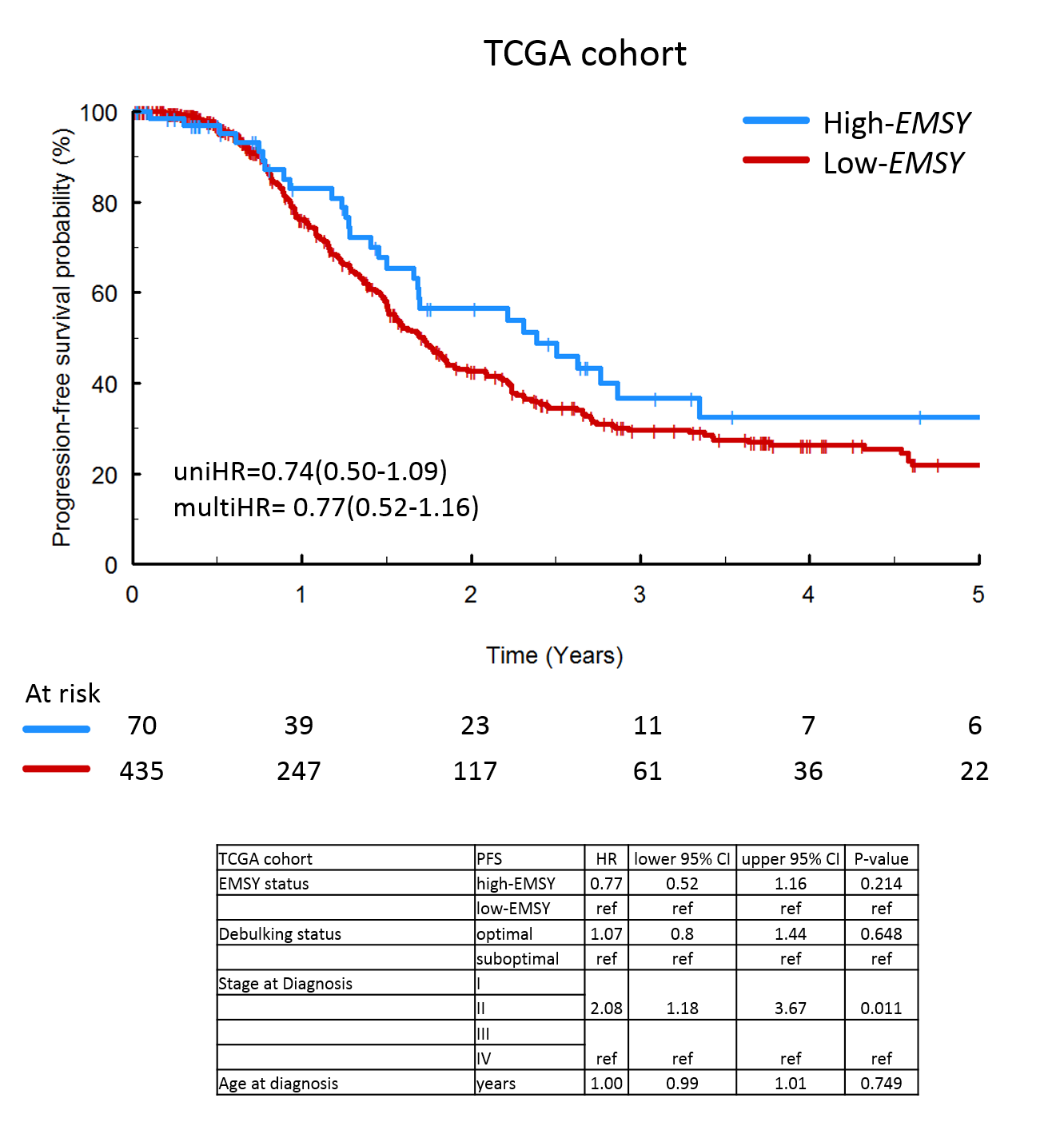

Supplement: Supplementary file 5 [file CNCR-125-2772-s005.tif]

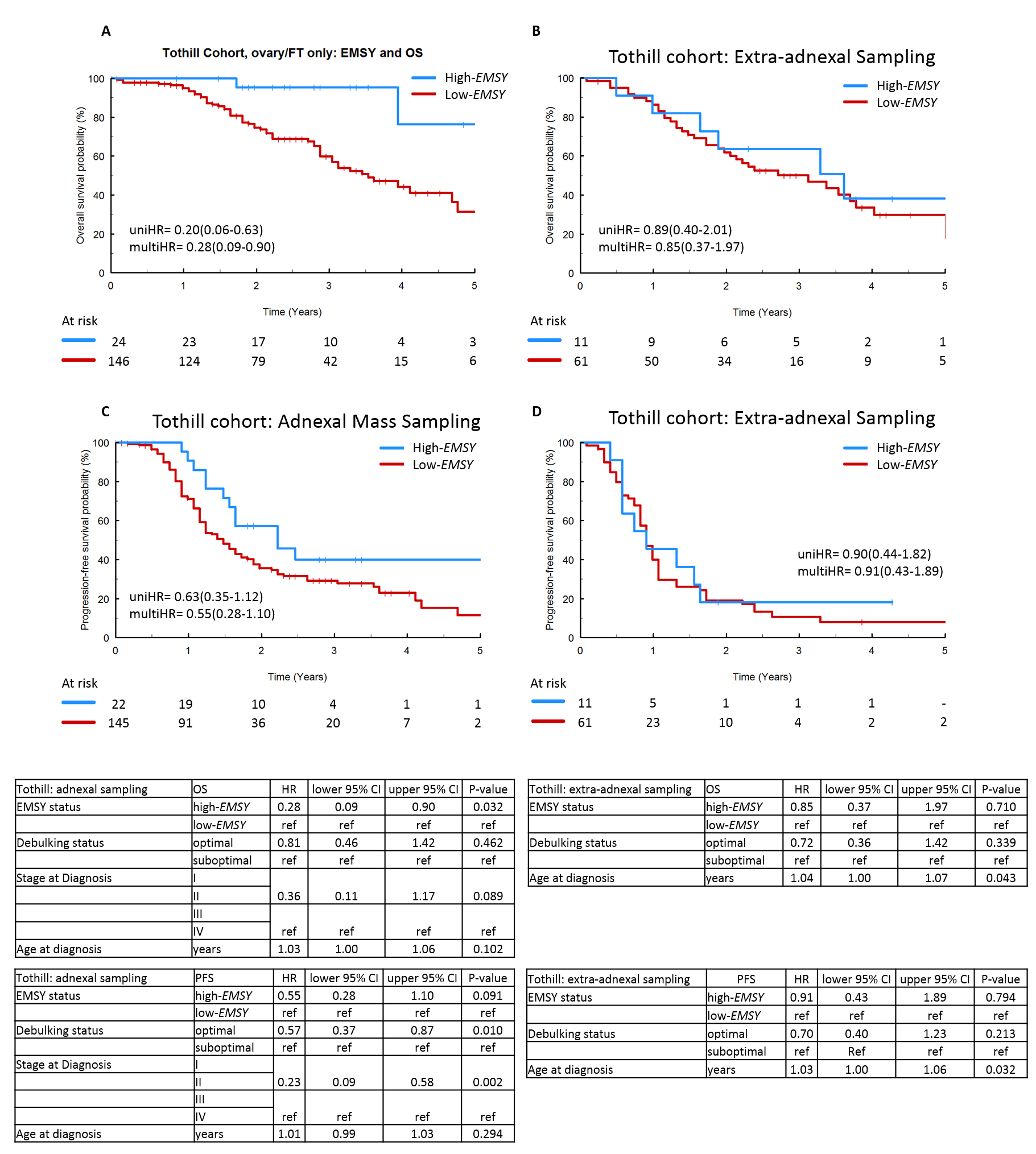

Supplement: Supplementary file 6 [file CNCR-125-2772-s006.tif]

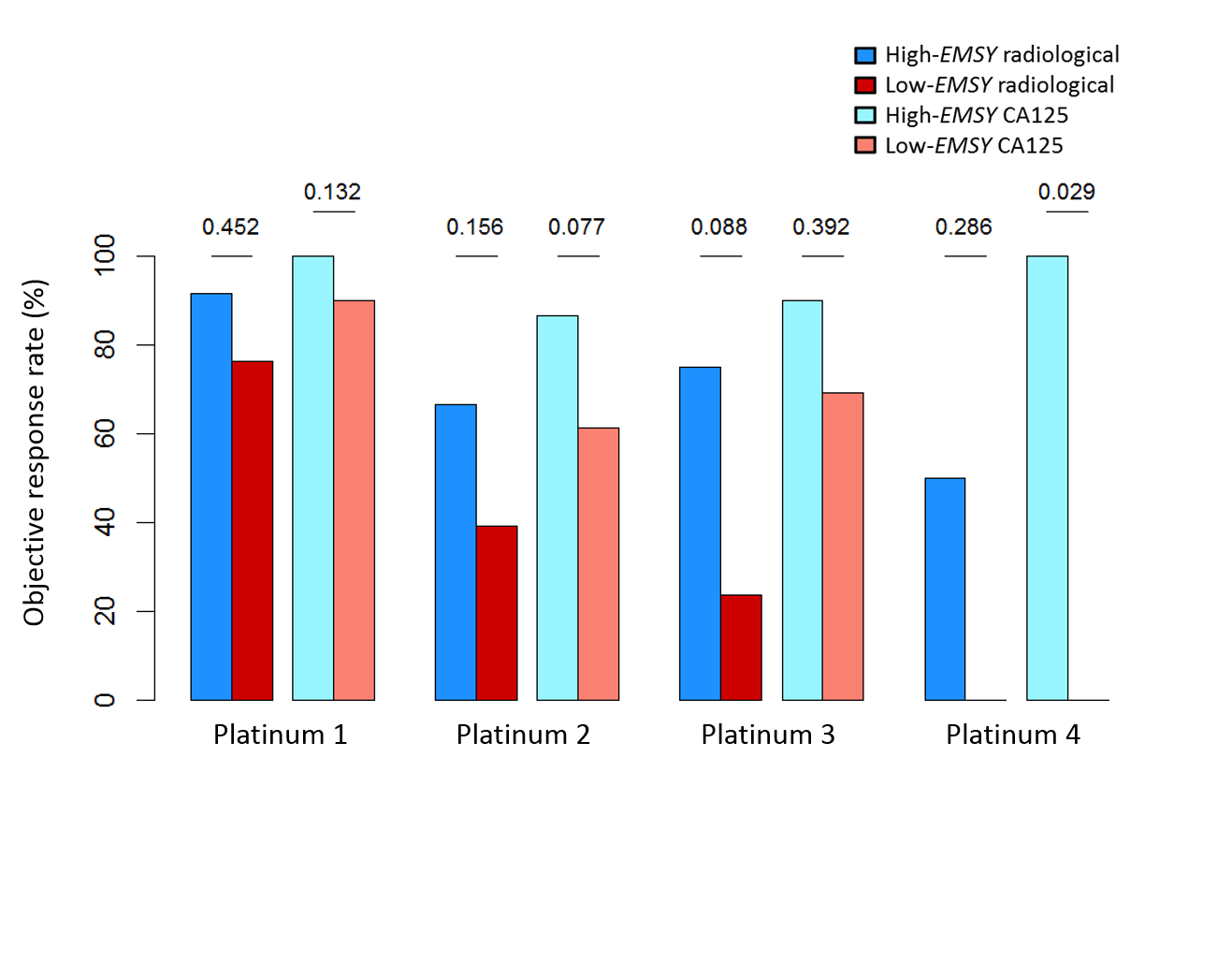

Supplement: Supplementary file 7 [file CNCR-125-2772-s007.tif]

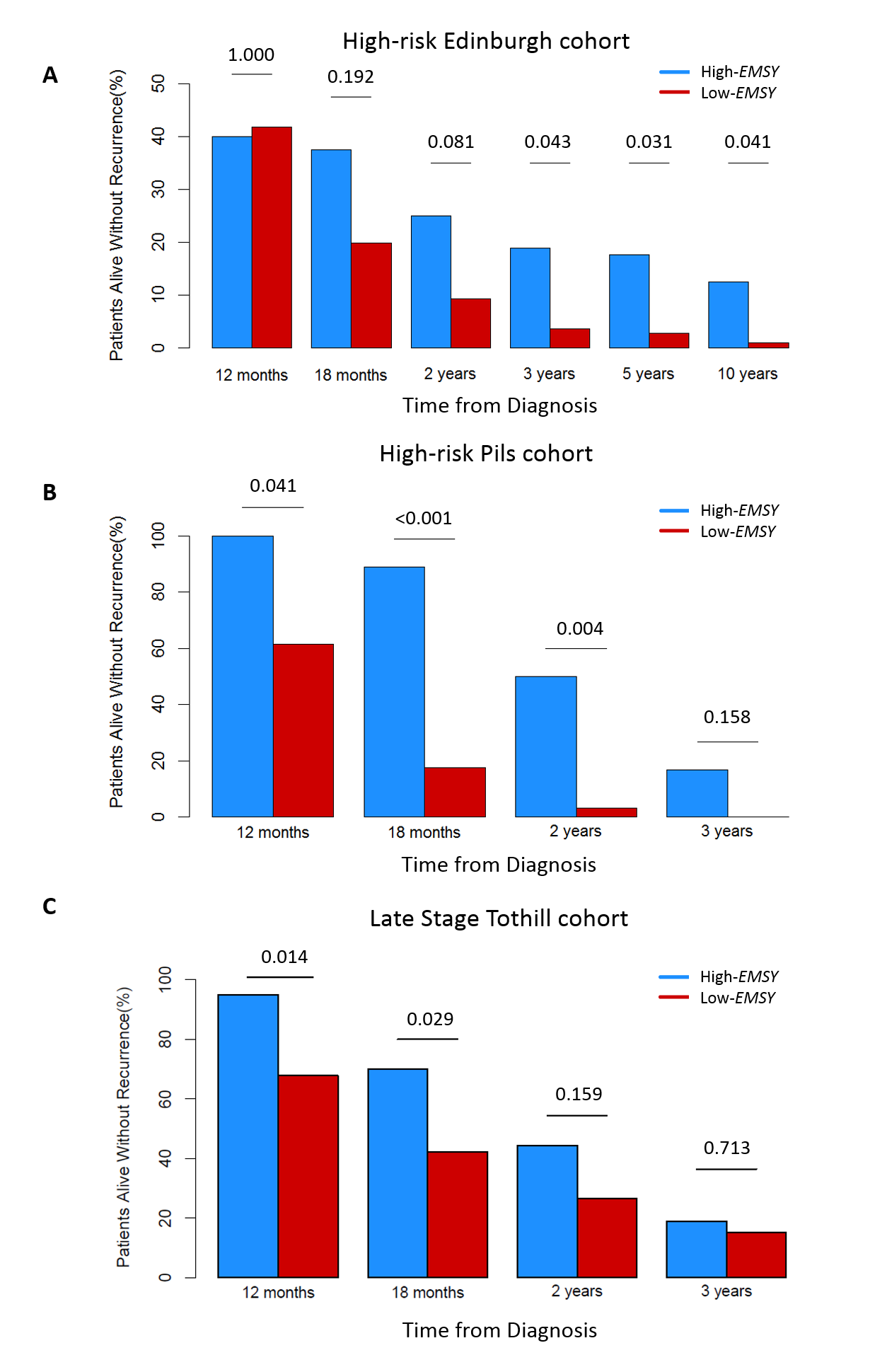

Supplement: Supplementary file 8 [file CNCR-125-2772-s008.tif]

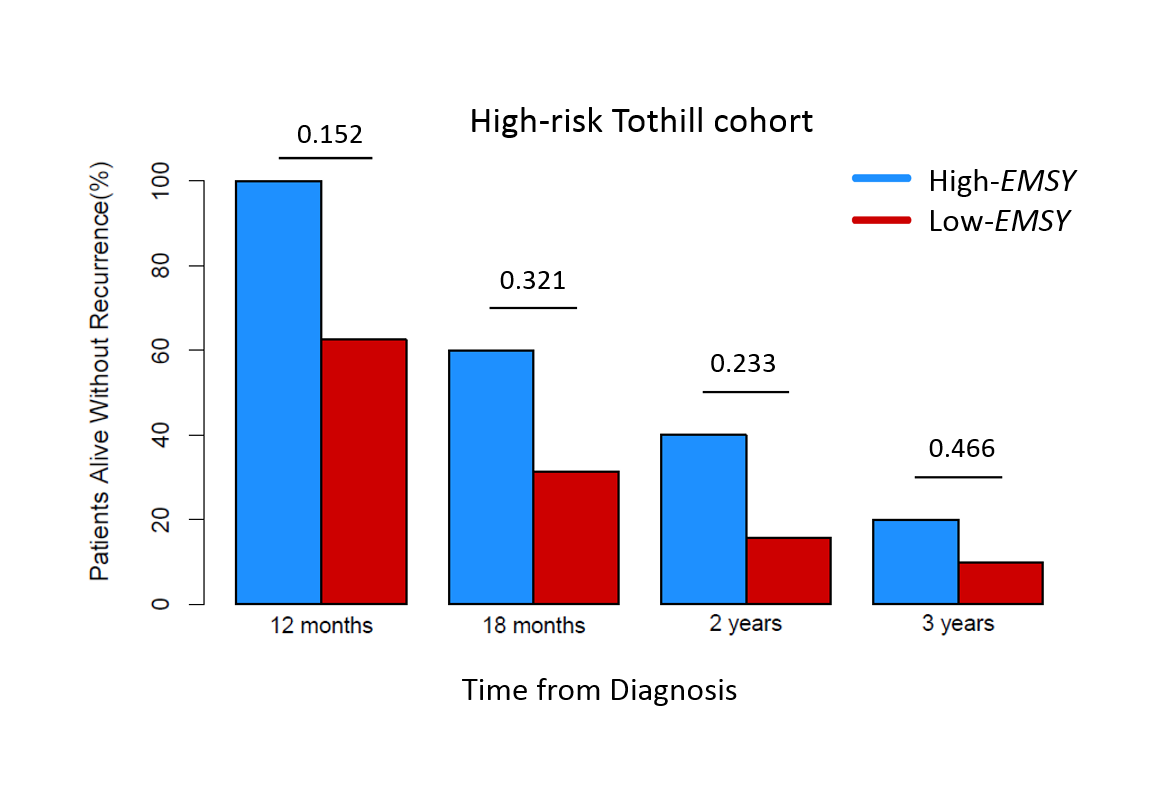

Supplement: Supplementary file 9 [file CNCR-125-2772-s009.tif]
