# Supplementary material for: High EMSY expression defines a BRCA‐like subgroup of high‐grade serous ovarian carcinoma with prolonged survival and hypersensitivity to platinum
Source: Cancer. 2019 Jun 2;125(16):2772–81. doi: 10.1002/cncr.32079 (PMC6771827; doi:10.1002/cncr.32079)
Supplement: Supplementary file 10 [file CNCR-125-2772-s010.docx]

CNCR_32079_e.Supporting Information_e.Table S1. *EMSY* expression in the Edinburgh cohort.

| Case | EMSY expression |
| --- | --- |
| HGSOC_1 | 4.6717 |
| HGSOC_2 | 3.7076 |
| HGSOC_3 | 4.3041 |
| HGSOC_4 | 5.0807 |
| HGSOC_5 | 3.4055 |
| HGSOC_6 | 4.4698 |
| HGSOC_7 | 3.6903 |
| HGSOC_8 | 4.1888 |
| HGSOC_9 | 3.1958 |
| HGSOC_10 | 3.7341 |
| HGSOC_11 | 4.0029 |
| HGSOC_12 | 3.7908 |
| HGSOC_13 | 4.0334 |
| HGSOC_14 | 3.8335 |
| HGSOC_15 | 4.4341 |
| HGSOC_16 | 4.1074 |
| HGSOC_17 | 4.0260 |
| HGSOC_18 | 4.0732 |
| HGSOC_19 | 3.1931 |
| HGSOC_20 | 3.9542 |
| HGSOC_21 | 3.1552 |
| HGSOC_22 | 3.6124 |
| HGSOC_23 | 3.3481 |
| HGSOC_24 | 3.2385 |
| HGSOC_25 | 4.4797 |
| HGSOC_26 | 4.6907 |
| HGSOC_27 | 3.4857 |
| HGSOC_28 | 3.8385 |
| HGSOC_29 | 4.3277 |
| HGSOC_30 | 3.6640 |
| HGSOC_31 | 4.8517 |
| HGSOC_32 | 3.1229 |
| HGSOC_33 | 4.7321 |
| HGSOC_34 | 4.6204 |
| HGSOC_35 | 4.4252 |
| HGSOC_36 | 4.7315 |
| HGSOC_37 | 3.7347 |
| HGSOC_38 | 7.3344 |
| HGSOC_39 | 4.8343 |
| HGSOC_40 | 3.9460 |
| HGSOC_41 | 4.2151 |
| HGSOC_42 | 4.4214 |
| HGSOC_43 | 3.5225 |
| HGSOC_44 | 5.0400 |
| HGSOC_45 | 4.7206 |
| HGSOC_46 | 5.5120 |
| HGSOC_47 | 4.5920 |
| HGSOC_48 | 4.3813 |
| HGSOC_49 | 3.2359 |
| HGSOC_50 | 3.2779 |
| HGSOC_51 | 4.0065 |
| HGSOC_52 | 3.4872 |
| HGSOC_53 | 4.7630 |
| HGSOC_54 | 4.0175 |
| HGSOC_55 | 4.0317 |
| HGSOC_56 | 4.4533 |
| HGSOC_57 | 3.7837 |
| HGSOC_58 | 7.1110 |
| HGSOC_59 | 4.4738 |
| HGSOC_60 | 5.9522 |
| HGSOC_61 | 3.6624 |
| HGSOC_62 | 2.7335 |
| HGSOC_63 | 4.5557 |
| HGSOC_64 | 4.1214 |
| HGSOC_65 | 4.0265 |
| HGSOC_66 | 4.6001 |
| HGSOC_67 | 3.9674 |
| HGSOC_68 | 4.1455 |
| HGSOC_69 | 4.8131 |
| HGSOC_70 | 4.3006 |
| HGSOC_71 | 4.6571 |
| HGSOC_72 | 5.0478 |
| HGSOC_73 | 4.8970 |
| HGSOC_74 | 4.3460 |
| HGSOC_75 | 4.3902 |
| HGSOC_76 | 3.9606 |
| HGSOC_77 | 4.5581 |
| HGSOC_78 | 4.5511 |
| HGSOC_79 | 4.3853 |
| HGSOC_80 | 3.7846 |
| HGSOC_81 | 3.1399 |
| HGSOC_82 | 4.4219 |
| HGSOC_83 | 3.6344 |
| HGSOC_84 | 4.2364 |
| HGSOC_85 | 4.9845 |
| HGSOC_86 | 4.1427 |
| HGSOC_87 | 4.9620 |
| HGSOC_88 | 4.9070 |
| HGSOC_89 | 4.6768 |
| HGSOC_90 | 4.3169 |
| HGSOC_91 | 4.2886 |
| HGSOC_92 | 4.2784 |
| HGSOC_93 | 3.2344 |
| HGSOC_94 | 3.3903 |
| HGSOC_95 | 4.1793 |
| HGSOC_96 | 4.6610 |
| HGSOC_97 | 3.8983 |
| HGSOC_98 | 3.4905 |
| HGSOC_99 | 4.5382 |
| HGSOC_100 | 3.3011 |
| HGSOC_101 | 3.0833 |
| HGSOC_102 | 3.1260 |
| HGSOC_103 | 4.4169 |
| HGSOC_104 | 4.2702 |
| HGSOC_105 | 4.6135 |
| HGSOC_106 | 4.1841 |
| HGSOC_107 | 3.9194 |
| HGSOC_108 | 3.9039 |
| HGSOC_109 | 4.4689 |
| HGSOC_110 | 4.7835 |
| HGSOC_111 | 5.3866 |
| HGSOC_112 | 3.9719 |
| HGSOC_113 | 4.1615 |
| HGSOC_114 | 4.5971 |
| HGSOC_115 | 4.1205 |
| HGSOC_116 | 4.4213 |
| HGSOC_117 | 3.9552 |
| HGSOC_118 | 4.9777 |
| HGSOC_119 | 4.7568 |
| HGSOC_120 | 4.3599 |
| HGSOC_121 | 3.7765 |
| HGSOC_122 | 3.6710 |
| HGSOC_123 | 4.8556 |
| HGSOC_124 | 4.9922 |
| HGSOC_125 | 5.7058 |
| HGSOC_126 | 4.9213 |
| HGSOC_127 | 4.6231 |
| HGSOC_128 | 5.1890 |
| HGSOC_129 | 3.8096 |
| HGSOC_130 | 4.2113 |
| HGSOC_131 | 3.1137 |
| HGSOC_132 | 4.7374 |
| HGSOC_133 | 3.5572 |
| HGSOC_134 | 3.9515 |
| HGSOC_135 | 3.9912 |
| HGSOC_136 | 4.7106 |
| HGSOC_137 | 3.8617 |
| HGSOC_138 | 4.9170 |
| HGSOC_139 | 4.4608 |
| HGSOC_140 | 3.6619 |
| HGSOC_141 | 4.1993 |
| HGSOC_142 | 4.1756 |
| HGSOC_143 | 4.8156 |
| HGSOC_144 | 4.0107 |
| HGSOC_145 | 4.3098 |
| HGSOC_146 | 4.5467 |
| HGSOC_147 | 4.7588 |
| HGSOC_148 | 3.7533 |
| HGSOC_149 | 4.7373 |
| HGSOC_150 | 4.4187 |
| HGSOC_151 | 3.7878 |
| HGSOC_152 | 4.1190 |
| HGSOC_153 | 3.8594 |
| HGSOC_154 | 6.4999 |
| HGSOC_155 | 4.6211 |
| HGSOC_156 | 4.2648 |
| HGSOC_157 | 4.7836 |
| HGSOC_158 | 4.3160 |
| HGSOC_159 | 4.3261 |
| HGSOC_160 | 4.4180 |
| HGSOC_161 | 5.0367 |
| HGSOC_162 | 5.0870 |
| HGSOC_163 | 3.7662 |
| HGSOC_164 | 4.5812 |
| HGSOC_165 | 4.3860 |
| HGSOC_166 | 4.3850 |
| HGSOC_167 | 3.8719 |
| HGSOC_168 | 4.4265 |
| HGSOC_169 | 3.6682 |
| HGSOC_170 | 4.8020 |
| HGSOC_171 | 3.8564 |
| HGSOC_172 | 4.0784 |
| HGSOC_173 | 3.7294 |
| HGSOC_174 | 3.6138 |
| HGSOC_175 | 3.7413 |
| HGSOC_176 | 3.8721 |
| HGSOC_177 | 4.1828 |
| HGSOC_178 | 3.4684 |
| HGSOC_179 | 3.7772 |
| HGSOC_180 | 3.6001 |
| HGSOC_181 | 4.2649 |
| HGSOC_182 | 3.5356 |
| HGSOC_183 | 3.4568 |
| HGSOC_184 | 3.8558 |
| HGSOC_185 | 3.7031 |
| HGSOC_186 | 3.4460 |
| HGSOC_187 | 3.6845 |
| HGSOC_188 | 3.7530 |
| HGSOC_189 | 4.4299 |
| HGSOC_190 | 4.2113 |
| HGSOC_191 | 4.3530 |
| HGSOC_192 | 3.2812 |
| HGSOC_193 | 4.2382 |
| HGSOC_194 | 3.8335 |
| HGSOC_195 | 4.7040 |
| HGSOC_196 | 4.1020 |
| HGSOC_197 | 4.1940 |
| HGSOC_198 | 5.1371 |
| HGSOC_199 | 4.2899 |
| HGSOC_200 | 4.1033 |
| HGSOC_201 | 3.5135 |
| HGSOC_202 | 3.4740 |
| HGSOC_203 | 4.3269 |
| HGSOC_204 | 3.9500 |
| HGSOC_205 | 4.2136 |
| HGSOC_206 | 4.1547 |
| HGSOC_207 | 4.9650 |
| HGSOC_208 | 3.5445 |
| HGSOC_209 | 4.4893 |
| HGSOC_210 | 4.3700 |
| HGSOC_211 | 4.5027 |
| HGSOC_212 | 4.2414 |
| HGSOC_213 | 3.9527 |
| HGSOC_214 | 4.3579 |
| HGSOC_215 | 3.6560 |
| HGSOC_216 | 4.0088 |
| HGSOC_217 | 3.1029 |
| HGSOC_218 | 4.1084 |
| HGSOC_219 | 3.9619 |
| HGSOC_220 | 3.2954 |
| HGSOC_221 | 4.0455 |
| HGSOC_222 | 3.7540 |
| HGSOC_223 | 4.2764 |
| HGSOC_224 | 4.1609 |
| HGSOC_225 | 3.7836 |
| HGSOC_226 | 3.0595 |
| HGSOC_227 | 3.9275 |
| HGSOC_228 | 3.6534 |
| HGSOC_229 | 4.0849 |
| HGSOC_230 | 4.2643 |
| HGSOC_231 | 4.2596 |
| HGSOC_232 | 3.9780 |
| HGSOC_233 | 4.1693 |
| HGSOC_234 | 4.3585 |
| HGSOC_235 | 4.3291 |
| HGSOC_236 | 3.7414 |
| HGSOC_237 | 4.9345 |
| HGSOC_238 | 3.8230 |
| HGSOC_239 | 4.0720 |
| HGSOC_240 | 3.5850 |
| HGSOC_241 | 3.3560 |
| HGSOC_242 | 5.8100 |
| HGSOC_243 | 4.0910 |
| HGSOC_244 | 4.0828 |
| HGSOC_245 | 4.2221 |
| HGSOC_246 | 4.2631 |
| HGSOC_247 | 2.7555 |
| HGSOC_248 | 3.7392 |
| HGSOC_249 | 4.6591 |
| HGSOC_250 | 4.1476 |
| HGSOC_251 | 3.8529 |
| HGSOC_252 | 3.2130 |
| HGSOC_253 | 3.6004 |
| HGSOC_254 | 3.5262 |
| HGSOC_255 | 3.0553 |
| HGSOC_256 | 4.2910 |
| HGSOC_257 | 3.9117 |
| HGSOC_258 | 3.2278 |
| HGSOC_259 | 4.0496 |
| HGSOC_260 | 4.0569 |
| HGSOC_261 | 3.7058 |
| HGSOC_262 | 3.6439 |
| HGSOC_263 | 4.2825 |
| HGSOC_264 | 3.3464 |
| HGSOC_265 | 3.7404 |

CNCR_32079_e.Supporting Information_e.Table S2. Correlation of six probes mapping to *EMSY* (RefSeq transcript NM_020193.3) used to calculate *EMSY* expression

| Spearman's rank | | rho | | | | | |
| --- | --- | --- | --- | --- | --- | --- | --- |
|  |  | Probe #1 | Probe #2 | Probe #3 | Probe #4 | Probe #5 | Probe #6 |
| P-value | Probe #1 |  | 0.232 | 0.252 | 0.205 | 0.283 | 0.294 |
|  | Probe #2 | 0.00014 |  | 0.348 | 0.344 | 0.217 | 0.305 |
|  | Probe #3 | 0.00003 | <0.00001 |  | 0.522 | 0.396 | 0.475 |
|  | Probe #4 | 0.00079 | <0.00001 | <0.00001 |  | 0.305 | 0.557 |
|  | Probe #5 | <0.00001 | 0.00037 | <0.00001 | <0.00001 |  | 0.437 |
|  | Probe #6 | <0.00001 | <0.00001 | <0.00001 | <0.00001 | <0.00001 |  |

CNCR_32079_e.Supporting Information_e.Table S3. Multivariable tables for (S3.1) Edinburgh cohort OS; (S3.2) Edinburgh cohort PFS; (S3.3) MRC ICON7 cohort OS; (S3.4) MRC ICON7 high-*EMSY* patients; (S3.5) MRC ICON7 control arm OS; (S3.6) Pils cohort OS; (S3.7) Pils cohort PFS; (S3.8) Mateescu cohort OS; (S3.9) Mateescu cohort PFS; (S3.10) Tothill cohort OS; (S3.11) Tothill cohort PFS; (S3.12) TCGA stage III/IV patients PFS.

S3.1

| Edinburgh Cohort | OS | HR | lower 95% CI | upper 95% CI | P-value |
| --- | --- | --- | --- | --- | --- |
| *EMSY* status | high-*EMSY* | 0.58 | 0.38 | 0.88 | 0.011 |
|  | low-*EMSY* | ref | ref | ref | ref |
| Debulking status | <2cm* | 0.64 | 0.46 | 0.88 | 0.006 |
|  | ≥2cm | ref | ref | ref | ref |
| Stage at Diagnosis | I | 0.38 | 0.23 | 0.63 | <0.001 |
|  | II |  |  |  |  |
|  | III | ref | ref | ref | ref |
|  | IV |  |  |  |  |
| Age at diagnosis | years | 1.01 | 1.00 | 1.02 | 0.175 |

S3.2

| Edinburgh Cohort | PFS | HR | lower 95% CI | upper 95% CI | P-value |
| --- | --- | --- | --- | --- | --- |
| *EMSY* status | high-*EMSY* | 0.62 | 0.40 | 0.96 | 0.030 |
|  | low-*EMSY* | ref | ref | ref | ref |
| Debulking status | <2cm* | 0.60 | 0.43 | 0.84 | 0.003 |
|  | ≥2cm | ref | ref | ref | ref |
| Stage at Diagnosis | I | 0.32 | 0.19 | 0.54 | <0.001 |
|  | II |  |  |  |  |
|  | III | ref | ref | ref | ref |
|  | IV |  |  |  |  |
| Age at diagnosis | years | 1.01 | 1.00 | 1.03 | 0.167 |

S3.3

| MRC ICON7 Cohort | OS | HR | lower 95% CI | upper 95% CI | P-value |
| --- | --- | --- | --- | --- | --- |
| *EMSY* status | high-*EMSY* | 0.46 | 0.23 | 0.91 | 0.025 |
|  | low-*EMSY* | ref | ref | ref | ref |
| Debulking status | <1cm | 0.52 | 0.38 | 0.71 | <0.001 |
|  | >1cm | ref | ref | ref | ref |
| Stage at Diagnosis | I | 0.28 | 0.15 | 0.52 | <0.001 |
|  | II |  |  |  |  |
|  | III | ref | ref | ref | ref |
|  | IV |  |  |  |  |
| Trial arm | bevacizumab | 1.34 | 0.99 | 1.81 | 0.055 |
|  | placebo | ref | ref | ref | ref |
| Age at diagnosis | years | 10.3 | 1.01 | 1.04 | <0.001 |

S3.4

| MRC ICON7 high-*EMSY* | OS | HR | lower 95% CI | upper 95% CI | P-value |
| --- | --- | --- | --- | --- | --- |
| Trial arm | bevacizumab | 11.78 | 1.31 | 106.32 | 0.028 |
|  | placebo | ref | ref | ref | ref |
| Debulking status | optimal | 0.7 | 0.16 | 3.18 | 0.646 |
|  | suboptimal | ref | ref | ref | ref |
| Stage at Diagnosis | II | 0.00 | 0.00 | Inf | 0.999 |
|  | III | ref | ref | ref | ref |
|  | IV | 0.21 | 0.03 | 1.42 | 0.109 |
| Age at diagnosis |  | 1.04 | 0.98 | 1.11 | 0.208 |

S3.5

| MRC ICON7 control cohort | OS | HR | lower 95% CI | upper 95% CI | P-value |
| --- | --- | --- | --- | --- | --- |
| *EMSY* status | high-*EMSY* | 0.12 | 0.02 | 0.83 | 0.032 |
|  | low-*EMSY* | ref | ref | ref | ref |
| Debulking status | optimal | 0.43 | 0.27 | 0.68 | <0.001 |
|  | suboptimal | ref | ref | ref | ref |
| Stage at Diagnosis | I | 0.30 | 0.12 | 0.76 | 0.011 |
|  | II |  |  |  |  |
|  | III | ref | ref | ref | ref |
|  | IV |  |  |  |  |
| Age at diagnosis | years | 1.02 | 1.00 | 1.04 | 0.039 |

S3.6

| Pils cohort | OS | HR | lower 95% CI | upper 95% CI | P-value |
| --- | --- | --- | --- | --- | --- |
| *EMSY* status | high-*EMSY* | 0.27 | 0.08 | 0.87 | 0.028 |
|  | low-*EMSY* | ref | ref | ref | ref |
| Debulking status | optimal | 0.64 | 0.35 | 1.19 | 0.163 |
|  | suboptimal | ref | ref | ref | ref |
| Stage at Diagnosis | I | 1.81 | 0.43 | 7.71 | 0.421 |
|  | II |  |  |  |  |
|  | III | ref | ref | ref | ref |
|  | IV |  |  |  |  |
| Age at diagnosis | years | 1.04 | 1.01 | 1.07 | 0.004 |

S3.7

| Mateescu cohort | OS | HR | lower 95% CI | upper 95% CI | P-value |
| --- | --- | --- | --- | --- | --- |
| *EMSY* status | high-*EMSY* | 0.43 | 0.18 | 0.99 | 0.048 |
|  | low-*EMSY* | ref | ref | ref | ref |
| Stage at Diagnosis | I | 0.49 | 0.23 | 1.05 | 0.066 |
|  | II |  |  |  |  |
|  | III | ref | ref | ref | ref |
|  | IV |  |  |  |  |

S3.8

| Pils cohort | PFS | HR | lower 95% CI | upper 95% CI | P-value |
| --- | --- | --- | --- | --- | --- |
| *EMSY* status | high-*EMSY* | 0.52 | 0.29 | 0.92 | 0.026 |
|  | low-*EMSY* | ref | ref | ref | ref |
| Debulking status | optimal | 0.50 | 0.32 | 0.76 | 0.001 |
|  | suboptimal | ref | ref | ref | ref |
| Stage at Diagnosis | I | 0.62 | 0.15 | 2.57 | 0.511 |
|  | II |  |  |  |  |
|  | III | ref | ref | ref | ref |
|  | IV |  |  |  |  |
| Age at diagnosis | years | 1.02 | 1.00 | 1.04 | 0.053 |

S3.9

| Mateescu cohort | PFS | HR | lower 95% CI | upper 95% CI | P-value |
| --- | --- | --- | --- | --- | --- |
| *EMSY* status | high-*EMSY* | 0.59 | 0.27 | 1.25 | 0.168 |
|  | low-*EMSY* | ref | ref | ref | ref |
| Stage at Diagnosis | I | 0.45 | 0.22 | 0.93 | 0.032 |
|  | II |  |  |  |  |
|  | III | ref | ref | ref | ref |
|  | IV |  |  |  |  |

S3.10

| Tothill cohort | OS | HR | lower 95% CI | upper 95% CI | P-value |
| --- | --- | --- | --- | --- | --- |
| *EMSY* status | high-*EMSY* | 0.60 | 0.32 | 1.13 | 0.112 |
|  | low-*EMSY* | ref | ref | ref | ref |
| Debulking status | optimal | 0.67 | 0.44 | 1.01 | 0.056 |
|  | suboptimal | ref | ref | ref | ref |
| Stage at Diagnosis | I | 0.32 | 0.10 | 1.02 | 0.055 |
|  | II |  |  |  |  |
|  | III | ref | ref | ref | ref |
|  | IV |  |  |  |  |
| Age at diagnosis | years | 1.03 | 1.01 | 1.05 | 0.016 |

S3.11

| Tothill cohort | PFS | HR | lower 95% CI | upper 95% CI | P-value |
| --- | --- | --- | --- | --- | --- |
| *EMSY* status | high-*EMSY* | 0.63 | 0.39 | 1.04 | 0.072 |
|  | low-*EMSY* | ref | ref | ref | ref |
| Debulking status | optimal | 0.56 | 0.40 | 0.79 | 0.001 |
|  | suboptimal | ref | ref | ref | ref |
| Stage at Diagnosis | I | 0.21 | 0.08 | 0.51 | 0.001 |
|  | II |  |  |  |  |
|  | III | ref | ref | ref | ref |
|  | IV |  |  |  |  |
| Age at diagnosis | years | 1.02 | 1.00 | 1.03 | 0.054 |

S3.12

| TCGA cohort stage III/IV | PFS | HR | lower 95% CI | upper 95% CI | P-value |
| --- | --- | --- | --- | --- | --- |
| *EMSY* status | high-*EMSY* | 0.68 | 0.45 | 1.04 | 0.076 |
|  | low-*EMSY* | ref | ref | ref | ref |
| Debulking status | optimal | 1.09 | 0.81 | 1.47 | 0.576 |
|  | suboptimal | ref | ref | ref | ref |
| Stage at Diagnosis | III | ref | ref | ref | ref |
|  | IV | 0.87 | 0.58 | 1.3 | 0.501 |
| Age at diagnosis | years | 1 | 0.99 | 1.02 | 0.617 |

CNCR_32079_e.Supporting Information_e.Table S4. Platinum response rates stratified by type of platinum-containing regime.

| Platinum exposure | Response Type | Therapy Class | EMSY status | n evaluable | CR | PR/ GCIG50% | no change | PD | CR rate (%) | PR rate (%) | P-value, high vs low EMSY CR rate | P-value high vs low EMSY ORR rate |
| --- | --- | --- | --- | --- | --- | --- | --- | --- | --- | --- | --- | --- |
| 1 | CA125 | Other platinum combination | high-EMSY | 0 | 0 | 0 | 0 | 0 | NA | NA | NA | NA |
|  |  |  | low-EMSY | 4 | 3 | 0 | 1 | 0 | 75.0 | 75.0 |  |  |
|  |  | Single-agent platinum | high-EMSY | 11 | 9 | 2 | 0 | 0 | 81.8 | 100.0 | 0.023 | 0.350 |
|  |  |  | low-EMSY | 90 | 39 | 38 | 10 | 3 | 43.3 | 85.6 |  |  |
|  |  | Platinum-taxane combination | high-EMSY | 14 | 13 | 1 | 0 | 0 | 92.9 | 100.0 | 0.162 | 1.000 |
|  |  |  | low-EMSY | 55 | 40 | 14 | 0 | 1 | 72.7 | 98.2 |  |  |
|  | Radiological | Other platinum combination | high-EMSY | 0 | 0 | 0 | 0 | 0 | NA | NA | NA | NA |
|  |  |  | low-EMSY | 6 | 2 | 2 | 0 | 2 | 33.3 | 66.7 |  |  |
|  |  | Single-agent platinum | high-EMSY | 4 | 1 | 3 | 0 | 0 | 25.0 | 100.0 | 1.000 | 0.302 |
|  |  |  | low-EMSY | 40 | 11 | 16 | 5 | 8 | 27.5 | 67.5 |  |  |
|  |  | Platinum-taxane combination | high-EMSY | 8 | 6 | 1 | 0 | 1 | 75.0 | 87.5 | 0.259 | 1.000 |
|  |  |  | low-EMSY | 34 | 17 | 13 | 2 | 2 | 50.0 | 88.2 |  |  |
| 2 | CA125 | Other platinum combination | high-EMSY | 2 | 1 | 0 | 1 | 0 | 50.0 | 50.0 | 0.517 | 0.506 |
|  |  |  | low-EMSY | 12 | 2 | 7 | 2 | 1 | 16.7 | 75.0 |  |  |
|  |  | Single-agent platinum | high-EMSY | 12 | 7 | 4 | 0 | 1 | 58.3 | 91.7 | 0.010 | 0.026 |
|  |  |  | low-EMSY | 66 | 13 | 25 | 27 | 1 | 19.7 | 57.6 |  |  |
|  |  | Platinum-taxane combination | high-EMSY | 1 | 0 | 1 | 0 | 0 | 0.0 | 100.0 | 0.333 | 1.000 |
|  |  |  | low-EMSY | 2 | 2 | 0 | 0 | 0 | 100.0 | 100.0 |  |  |
|  | Radiological | Other platinum combination | high-EMSY | 0 | 0 | 0 | 0 | 0 | NA | NA | NA | NA |
|  |  |  | low-EMSY | 7 | 1 | 3 | 2 | 1 | 14.3 | 57.1 |  |  |
|  |  | Single-agent platinum | high-EMSY | 7 | 3 | 2 | 2 | 0 | 42.9 | 71.4 | 0.059 | 0.093 |
|  |  |  | low-EMSY | 54 | 6 | 12 | 17 | 19 | 11.1 | 33.3 |  |  |
|  |  | Platinum-taxane combination | high-EMSY | 2 | 1 | 0 | 0 | 1 | 50.0 | 50.0 | 1.000 | 0.400 |
|  |  |  | low-EMSY | 3 | 1 | 2 | 0 | 0 | 33.3 | 100.0 |  |  |
| 3 | CA125 | Other platinum combination | high-EMSY | 3 | 0 | 2 | 1 | 0 | 0.0 | 66.7 | 1.000 | 0.491 |
|  |  |  | low-EMSY | 8 | 0 | 7 | 1 | 0 | 0.0 | 87.5 |  |  |
|  |  | Single-agent platinum | high-EMSY | 6 | 4 | 2 | 0 | 0 | 66.7 | 100.0 | 0.150 | 0.130 |
|  |  |  | low-EMSY | 18 | 5 | 6 | 7 | 0 | 27.8 | 61.1 |  |  |
|  |  | Platinum-taxane combination | high-EMSY | 1 | 0 | 1 | 0 | 0 | 0.0 | 100.0 | NA | NA |
|  |  |  | low-EMSY | 0 | 0 | 0 | 0 | 0 | NA | NA |  |  |
|  | Radiological | Other platinum combination | high-EMSY | 2 | 0 | 1 | 1 | 0 | 0.0 | 50.0 | 1.000 | 1.000 |
|  |  |  | low-EMSY | 2 | 0 | 0 | 2 | 0 | 0.0 | 0.0 |  |  |
|  |  | Single-agent platinum | high-EMSY | 2 | 2 | 0 | 0 | 0 | 100.0 | 100.0 | 0.022 | 0.110 |
|  |  |  | low-EMSY | 15 | 1 | 3 | 4 | 7 | 6.7 | 26.7 |  |  |
|  |  | Platinum-taxane combination | high-EMSY | 0 | 0 | 0 | 0 | 0 | NA | NA | NA | NA |
|  |  |  | low-EMSY | 0 | 0 | 0 | 0 | 0 | NA | NA |  |  |
