# Supplementary material for: High EMSY expression defines a BRCA‐like subgroup of high‐grade serous ovarian carcinoma with prolonged survival and hypersensitivity to platinum
Source: Cancer. 2019 Jun 2;125(16):2772–81. doi: 10.1002/cncr.32079 (PMC6771827; doi:10.1002/cncr.32079)
Supplement: Supplementary file 11 [file CNCR-125-2772-s011.docx]

**Supporting Information Figure Legends**

CNCR_32079_e.Supporting Information_e.Figure S1. Distribution of *EMSY* expression across transcriptomically characterised HGSOC cohorts.

CNCR_32079_e.Supporting Information_e.Figure S2. Identification of the threshold for *EMSY* overexpression using cut-point analysis of univariable survival within the Edinburgh cohort. Points indicate HR at the respective percentile expression threshold, with tails indicating corresponding 95% confidence intervals.

CNCR_32079_e.Supporting Information_e.Figure S3. PFS within the MRC ICON7 cohort.

CNCR_32079_e.Supporting Information_e.Figure S4. OS in the top 14% of EMSY expressers in the MRC ICON7 control arm.

CNCR_32079_e.Supporting Information_e.Figure S5. PFS of high-*EMSY* patients in the TCGA HGSOC cohort.

CNCR_32079_e.Supporting Information_e.Figure S6. Interaction between sampling site (primary mass versus extra-adnexal sampling) and prognostic impact of high *EMSY* expression within the Tothill cohort. (A) OS in high-*EMSY* HGSOC from adnexal specimens; (B) OS in high-*EMSY* HGSOC from extra-adnexal specimens; (C) PFS in high-*EMSY* HGSOC from adnexal specimens; (D) PFS in high-*EMSY* HGSOC from extra-adnexal specimens.

CNCR_32079_e.Supporting Information_e.Figure S7. Radiological and CA125 tumour marker objective response rate (complete response plus partial response) to platinum-containing chemotherapy.

CNCR_32079_e.Supporting Information_e.Figure S8. Long-term clinical outcome of high-*EMSY* patients in the context of otherwise poor prognosis. Proportion of high-*EMSY* patients alive without recurrence within the late stage suboptimally debulked (A) Edinburgh and (B) Pils cohorts, and stage III/IV Tothill cohort (C). Note: discrete variability in total patient numbers is due to a minority of patients being non-evaluable for PFS and subsequently experiencing OS events, preventing inclusion of these patients in the analysis preceding the OS event time point.

CNCR_32079_e.Supporting Information_e.Figure S9. Proportion of patients alive without disease recurrence in the advanced stage suboptimally debulked Tothill HGSOC cohort.
